# The Neural Cost of Language Processing: Broader Network Recruitment in Adults With Developmental Language Disorder

**DOI:** 10.1162/NOL.a.277

**Published:** 2026-08-28

**Authors:** Noelle T. Abbott, Linnea Beasley, Gabriel J. Cler

**Affiliations:** Department of Speech and Hearing Sciences, University of Washington, Seattle, WA, USA

**Keywords:** adults, developmental language disorder (DLD), fMRI, language localizer, language network, speech comprehension

## Abstract

Developmental language disorder (DLD) is a neurodevelopmental disorder characterized by persistent difficulties in language comprehension and production, despite otherwise typical cognitive abilities. Although DLD is most commonly studied in children, it often persists into adulthood, yet little is known about the neural substrates underlying language abilities in adults with DLD. To address this gap, we examined 50 adults with (*n* = 23) and without (*n* = 27) DLD using an fMRI language localizer paradigm. Participants passively listened to intelligible and acoustically degraded speech to assess both lower-level auditory processing and higher-level language comprehension. We also administered standardized language assessments to relate behavioral performance to neural activation patterns. Results indicated that auditory processing in DLD is largely intact, as both groups showed comparable activation in bilateral auditory cortices during degraded speech. In contrast, during comprehension of intact speech, adults with DLD recruited additional domain-general regions, including bilateral midline, frontal, and parietal areas. This pattern suggests compensatory or effortful engagement of broader cognitive systems to support comprehension, even during a very simple task. Critically, participants whose activation patterns more closely resembled canonical left-lateralized language networks demonstrated stronger performance on standardized language measures, implying that diffuse network recruitment may come at a cognitive cost. Overall, these findings indicate that while adults with DLD can achieve functional language comprehension, they do so through altered neural pathways.

## INTRODUCTION

Developmental language disorder (DLD; sometimes referred to as specific language impairment) is a neurodevelopmental disorder that is broadly characterized by language problems that cannot be explained by other factors or diagnoses, such as hearing differences or autism spectrum disorder (Bishop, 2017; Bishop et al., 2017). DLD impacts the ability to comprehend and produce spoken, written, or signed language, but the extent of impact can vary from person to person. DLD is a prevalent disorder that affects around 7% of the population (Norbury et al., 2016; Tomblin et al., 1997), which means that two children in an average-sized classroom will experience functional limitations within and beyond academic settings (McGregor, 2020). Importantly, these limitations can extend into adulthood. Adults with DLD often experience greater economic instability, fewer social relationships, and poorer mental health outcomes than their peers with typical language development (TD), and a large proportion of adults who are incarcerated would meet criteria for DLD (Clegg et al., 2005; Conti-Ramsden et al., 2017; Dubois et al., 2020; Winstanley et al., 2018). First-person accounts of DLD in adulthood highlight ongoing challenges with language comprehension and production that impact professional opportunities and overall well-being (Hirn, 2023; Orrego et al., 2023). Findings such as these underscore the importance of having a comprehensive understanding of DLD across the lifespan, particularly as children with DLD become adults with DLD who have to navigate increasingly complex language environments.

Despite the challenges faced by those with DLD and the pervasiveness of the disorder, relatively little is understood about the connection between language abilities and neural differences in children, and how or whether neural differences persist into adulthood. One reason for this limitation is the small number of neuroimaging studies that have been conducted in this population. Abbott and Love (2023) reviewed the existing neuroimaging literature with the goal of identifying neural differences consistently implicated in DLD but found limited consensus across studies, likely due to differences in participant age ranges, methods (e.g., structural vs. functional; different tasks), analytic approaches, and inclusion and exclusion criteria. The majority of extant studies focus on structural brain differences in children, with less research focused on functional brain differences. Important to the current study, only a handful of neuroimaging studies include adults with DLD, leaving a significant gap in understanding about the relationship between brain and behavior as children with DLD become adults with DLD (summarized below, see the Persistent Neural and Behavioral Differences in Adults With DLD section). This gap matters for forming a mechanistic framework that accounts for both brain and behavior in DLD.

Prominent theories of DLD, such as the Procedural Circuit Deficit Hypothesis (PDH) (Ullman & Pierpont, 2005; Ullman et al., 2020), make predictions about the neural systems underlying language abilities, but these predictions also need to capture brain–behavior dynamics beyond childhood and adolescence, particularly given that DLD persists into adulthood and that the neural systems underlying language continue to mature beyond childhood.

The brain changes rapidly in the first few years of life to support the explosion of newly learned skills and ideas (Bethlehem et al., 2022). Neuroimaging studies at this stage only capture a snapshot of the brain at a specific point in development, making it difficult to know whether neural differences reflect a neural system that has not caught up to that of its peers (implying that those differences may diminish over time) or a system that is developing in an aberrant or even compensatory manner (implying long-term alterations in structure and function). In early adulthood, neural systems are relatively stable, and language skills are no longer rapidly developing (Rosselli et al., 2014). The snapshot of the brain that we get from neuroimaging in adults may therefore provide a unique window into the complex dynamics between brain and language that could help fill in important gaps in knowledge about the brain and language in DLD. Thus, a necessary first step is establishing whether the brain regions supporting language processing differ between adults with DLD and adults with TD.

To form a foundation for understanding the brain regions consistently implicated in DLD, we start the section below with a brief summary of neuroimaging findings based on the review paper from Abbott and Love (2023), which, again, mainly focuses on structural MRI studies in children. We then move to a discussion of the limited neural and behavioral findings in adults with DLD.

### Neuroimaging Findings in Children With DLD

Even with methodological differences across studies, Abbott and Love (2023) identified some consistent patterns that have emerged in pediatric DLD literature. White-matter studies indicate altered microstructure in both dorsal and ventral language pathways, linked to receptive and expressive language performance (Roberts et al., 2014; Verhoeven et al., 2012; Verly et al., 2019; Vydrova et al., 2015). More recent work further highlights atypical myelin and cortical–subcortical microstructure in regions such as the frontal cortex, basal ganglia, thalamus, and hippocampus (Bahar et al., 2023; Cler et al., 2026; Doucet, Kruse, Mertens, et al., 2025; Krishnan et al., 2022; J. C. Lee et al., 2020; Ullman et al., 2024).

Relevant to the current study, functional alterations have been observed in language regions such as the inferior frontal gyrus (IFG), superior temporal gyrus (STG), middle temporal gyrus (MTG), and supramarginal gyrus (SMG), alongside gray matter differences in regions such as the pars triangularis portion of the IFG, planum temporale (PT), and posterior STG (Abbott & Love, 2023; Badcock et al., 2012; Bahar et al., 2023; Bryłka et al., 2025; De Fossé et al., 2004; Gauger et al., 1997; Jernigan et al., 1991; Kurth et al., 2018; Plante, 1991). Recent work from Doucet, Kruse, Eden, et al. (2025) also implicates altered functional connectivity within domain-general cognitive control, somatomotor, and default mode networks. Taken together, evidence from children with DLD points to widespread structural and functional differences across cortical and subcortical systems, but discrepancies across studies highlight the need for more work to clarify the precise nature of these differences.

### Persistent Neural and Behavioral Differences in Adults With DLD

Far fewer studies have examined adults with DLD, partly due to the absence of standardized diagnostic criteria beyond childhood (Fidler et al., 2011), leaving open questions about the extent to which neural differences observed in childhood persist into adulthood. Based on four studies, current neuroimaging findings in adults with DLD indicate morphological differences in IFG and other perisylvian structures, altered subcortical volumes (including caudate/putamen), and differences in white matter microstructure (J. C. Lee et al., 2013, 2020). Functional neuroimaging studies suggest that adults with DLD perform similarly to typically developing peers but show differences in neural activation during language tasks (Plante et al., 2006, 2017). Plante et al. (2017) found that adults with DLD could learn words from an unfamiliar language but showed hyperactivation in language regions, interpreted as reflecting greater cognitive effort. Plante et al. (2006) found group differences in brain activation during verbatim versus gist-level narrative processing, suggesting disrupted encoding strategies. Together, these findings indicate that adults with DLD may compensate for language difficulties by exerting greater effort or employing atypical neural strategies.

Likewise, behavioral research in adults with DLD converges on the persistence of difficulties. Adults with DLD continue to show weaknesses in sentence repetition, word learning, and lexical–semantic knowledge (Becker & McGregor, 2016; Botting, 2020; Del Tufo & Earle, 2020; Hall et al., 2017; Isaki & Plante, 1997; McGregor, Arbisi-Kelm, et al., 2017; McGregor, Gordon, et al., 2017; Poll et al., 2010, 2025; Records et al., 1995). Core clinical markers in childhood, such as nonword repetition, vocabulary knowledge, listening comprehension, and grammaticality judgments, remain relatively sensitive in adulthood (Fidler et al., 2011; Poll et al., 2010, 2025; Tomblin et al., 1992). Beyond these language challenges, adults with DLD also exhibit slower processing speed, persistent weaknesses in (verbal) working memory, and reduced efficiency in implicit and explicit learning (Grunow et al., 2006; McGregor et al., 2020; Miller & Poll, 2009; Plante et al., 2002; Poll & Martin, 2022; Poll & Miller, 2021; Poll et al., 2014, 2016; Richardson et al., 2006; von Koss Torkildsen et al., 2013), though not all learning is impacted (see Cler et al., 2024).

In summary, the adult behavioral literature points to enduring language and cognitive differences in DLD, with compensatory strategies and task-specific supports shaping the degree to which difficulties are observable. A small number of neuroimaging studies in adults with DLD indicate widespread neural differences, but the dearth of adult-focused work prevents a better understanding of the ways in which these neural markers contribute to language differences. The present study addresses this gap by examining neural activation during a passive language processing task in adults with DLD compared to adults with TD.

### Mapping the Core Language Network

One fruitful approach to investigating the functional neural architecture of language is the use of functional paradigms that reliably map the “core” language network. For example, Fedorenko and colleagues have developed robust language localizer tasks that consistently identify a left-lateralized network of frontal and temporal regions involved in higher-level language processing (Fedorenko et al., 2010, 2011; Scott et al., 2017). The language localizer method is highly reliable across participants with typical language and separates auditory processing from higher-level linguistic processing (Scott et al., 2017). The passive auditory-only localizer developed by Scott et al. (2017) further capitalizes on these benefits by eliminating the reading demands of the original task. This modification is particularly important for language-impaired populations. Rapid reading is cognitively demanding and may be disproportionately difficult for individuals with language impairments, potentially confounding neural activation patterns. A passive language task instead allows researchers to obtain clear, participant-specific maps of the language network across individuals who may differ in attention, reading ability, or task strategies, including those with DLD (Hernández et al., 2022; Poll et al., 2015; Shafer et al., 2007). The paradigm employed here requires no active task engagement (beyond listening) or literacy skills, minimizing the demands placed on the listener. This makes it well suited to address our foundational question of whether the language network differs in adults with DLD compared to adults with TD.

The use of functional language localizers has been applied to both typically developing (Malins et al., 2016) and neurodivergent populations, including individuals with epilepsy, dyslexia, and autism, providing critical insights into how the language network is organized across a range of disorders. For example, in pediatric epilepsy, passive language localizer paradigms have been validated against invasive gold-standard mapping techniques, demonstrating their reliability in identifying language regions even in very young or sedated patients (Szaflarski et al., 2017). Language localizers have also been used in dyslexia research to compare the functional organization of the cortical language network between typical and struggling readers, with research indicating that both groups engage the canonical left-lateralized frontotemporal language network, albeit sometimes with additional or redistributed activation patterns that may reflect compensatory processes (J. J. Lee, 2022). Likewise, the use of language localizers with individuals with autism has revealed atypical recruitment of left-hemisphere language regions (Jouravlev et al., 2020). Collectively, this work illustrates how functional localizers provide a powerful, task-independent way to identify the brain regions associated with typical and atypical language processing, though the paradigm is not without criticism (e.g., see Grodzinsky, 2010; Murphy & Woolnough, 2024).

### Current Study

In this study, we examined the language network activated by an auditory-only language localizer in adults with and without DLD. We compared neural activation patterns obtained while participants listened to intelligible and unintelligible speech in the MRI scanner. Given the relatively robust language skills in this cohort (e.g., all but one participant attended college and all participants communicated effectively throughout the duration of the study) and the simplicity of the task, we hypothesized that language networks would be very similar between groups. A whole-brain, data-driven approach was chosen given the near absence of prior work in this population. In addition, group-level comparisons of activation patterns can mask important individual (within-group) differences; thus, to better understand differences in activation patterns, we generated activation overlap maps that illustrate the number of participants that had significant activation within a brain region. Finally, we examined whether performance on a standardized language assessment was associated with activation within the language network, predicting a priori that higher scores would correspond to greater activation in left-hemisphere frontotemporal language regions. Given that the neural regions involved in language processing are fairly well characterized in neurotypical adults (Billot et al., 2024; Malik-Moraleda et al., 2022; Price, 2010; Shain & Fedorenko, 2025), investigating whether this network is altered in adults with DLD provides an important opportunity to reveal the neural underpinnings of DLD beyond childhood.

## METHODS

### Participants

Twenty-seven adults with TD (mean age = 21.2 ± 3.4 years; 22 women, five men) and 23 adults with DLD (mean age = 22.6 ± 4.8 years; 18 women, four men, one nonbinary person) participated in this study. Participants were recruited throughout the university area and wider community and completed a written initial screening to determine eligibility; recruitment processes are described in more detail in the DLD Recruitment Protocol section below. All participants were screened for normal hearing based on an audiological evaluation with tones between 250 and 8000 Hz at 25 dB and reported normal or corrected-to-normal vision. Participants, in either group (DLD or TD), could not have diagnoses of attention-deficit/hyperactivity disorder (ADHD), acquired brain injuries (including more than one mild concussion), or other developmental disorders such as autism or Down syndrome based on a self-report. Additionally, participants had to report being spoken to in English before 2 years of age and have a primary caregiver who primarily spoke English to them as a baby. Within our sample, all participants reported that their primary caregiver(s) always spoke to them in English, with the exception of two participants with TD who reported that their caregivers mostly spoke to them in English. Bilingual participants were included in both groups if the requirements for English were met. In the DLD group, there were three bilingual participants, and in the TD group, there were eight bilingual participants. Participants provided written informed consent to participate. The testing protocol was reviewed and approved based on the University of Washington Institutional Review Board guidelines, and participants were compensated monetarily for their time.

**Figure 1.**
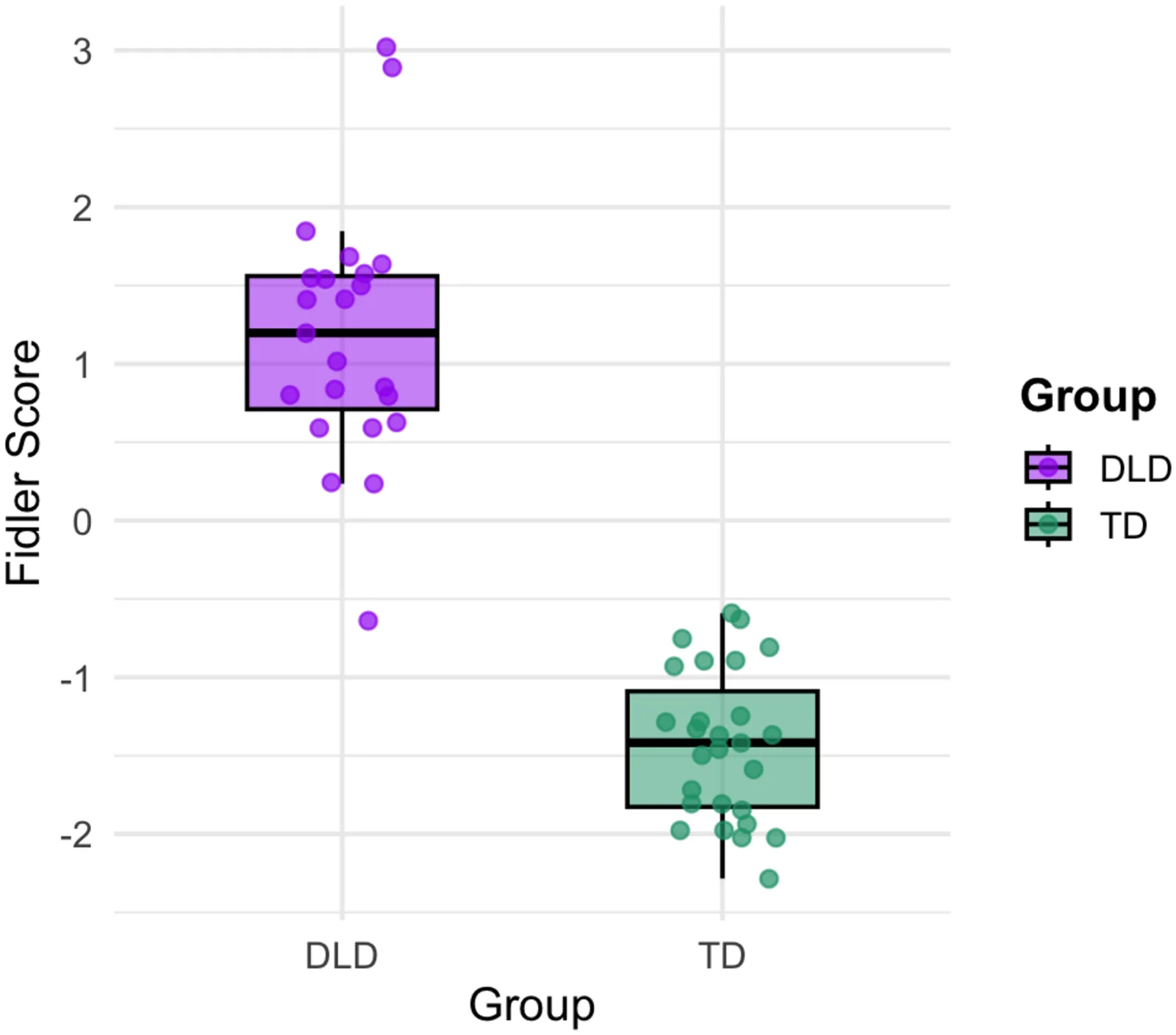
The distribution of Fidler scores between adults with developmental language disorder (DLD) and adults with typical language development (TD). According to the Fidler protocol, scores greater than 0 indicate inclusion in the DLD group, while scores less than 0 indicate inclusion in the TD group. Note that one participant with DLD scored within the TD range but was included in the DLD group due to having a clinical diagnosis of DLD from childhood.

After meeting inclusion and exclusion criteria above, group inclusion was determined by the procedure described by Fidler et al. (2011), modified for online assessment (Cler et al., 2024). Briefly, the Fidler protocol provides a weighted score based on a 15-word written spelling test consisting of irregularly spelled words of English and the Modified Token Test (an auditory following directions task). Scores from the two Fidler tasks are converted to a weighted score based on the two-test Fidler equation (6.5727 − 0.2184 * spelling − 0.1298 * token) shown to identify participants with a history of receiving speech-language services with a sensitivity of 80% and a specificity of 87% (Fidler et al., 2011). Scores below zero indicate TD, and scores above zero indicate impaired language consistent with a diagnosis of DLD, though we prioritized recruitment of participants who had scores above 0.5 or below −0.5. In addition to the Fidler protocol, all participants completed an extensive background questionnaire that included information about clinical diagnoses and language history. Notably, one participant with DLD scored within the TD range using the Fidler protocol; however, they were included in the DLD group as they had a childhood diagnosis of DLD from a licensed speech-language pathologist (SLP) and reported receiving treatment from an SLP until 9 years of age, along with accommodations through primary/secondary school, college, and graduate school. See Table 1 for participant demographic information and assessment scores. The distribution of Fidler scores can be seen in Figure 1.

**Table 1.** Participant demographics and assessment scores for adults with developmental language disorder (DLD) and adults with typical language development (TD).

Group	DLD (*n* = 23)	TD (*n* = 27)	Statistics
Age (range)	22.6 ± 4.8 (18–38)	21.2 ± 3.4 (18–32)	*t*(39.3) = 1.2, *p* = 0.24
Gender	4 men	5 men	Fisher’s exact, *p* = 0.85
	1 nonbinary person	22 women	
	18 women		
College Attended (*n*)	22 (96%)	27 (100%)	Fisher’s exact, *p* = 0.46
Fidler Score	1.18 ± 0.8	−1.44 ± 0.5	*t*(34.4) = 13.6, ***p* < 0.001**
TILLS Identification Core Score	94.5 ± 14.2	113.5 ± 6.6	*t*(29.9) = −5.9, ***p* < 0.001**
TILLS Sentence / Discourse Composite Score	94.0 ± 8.0	108.1 ± 10.7	*t*(47.3) = −5.3, ***p* < 0.001**
WASI-II Block Design	54.5 ± 8.6	57.7 ± 9.0	*t*(47.3) = −1.3, *p* = 0.20
WASI-II Matrix Reasoning	52.0 ± 8.9 (*n* = 22)	55.7 ± 9.3	*t*(44.7) = −1.4, *p* = 0.17

*Note*. Values are given for the mean ± standard deviation as standard scores for the TILLS composites and *T*-Scores for the WASI-II subtests. Values in **bold** indicate a significant difference between the DLD and TD groups. TILLS = Test of Integrated Language and Literacy Skills (Nelson et al., 2016); WASI-II = Wechsler Abbreviated Scale of Intelligence–Second Edition (Wechsler, 2018).

### DLD Recruitment Protocol

There are currently no well-established recruitment protocols for adults with DLD. Therefore, we adopted a broad recruitment strategy that captured a wide group of individuals who demonstrated difficulty with speech and language. Recruitment was achieved through two pathways: (1) recruiting anyone interested in participating in studies with a generic flyer and (2) using flyers that targeted participants who may have language differences and included questions such as “Have you ever received help for a learning disability?” and “Do you have trouble keeping up with conversations or lectures?” Participants with DLD were identified through both pathways.

Both flyers were distributed physically and online. Physical flyers were placed on bulletin boards at disability resource centers, tutoring centers, and writing centers, as well as throughout the college and city community in spaces such as libraries, coffee shops, community/cultural centers, and parks. Flyers were posted online largely through job boards for college students, although they were also distributed through social media. All flyers pointed potential participants to an initial screening form to determine eligibility. Eligible participants were sent a link to schedule an experimental session over Zoom to screen for their language abilities via the Fidler protocol modified for online assessment (Cler et al., 2024). Participants assigned to the DLD group sometimes reported having related diagnoses, including dyslexia, specific reading disorder, expressive/receptive language disorder, or specific learning disability, but some had no diagnoses. Participants with these diagnoses were not included in the TD group.

### Behavioral Measures

In addition to the Fidler screening and questionnaire forms, all participants completed the Test of Integrated Language and Literacy Skills (TILLS) (Nelson et al., 2016) to measure a range of linguistic and non-linguistic skills. Importantly, the TILLS has been age norm-referenced up to 18 years but has not been well validated for adults over 18 years of age. While assessments such as the Clinical Evaluation of Language Fundamentals, Fifth Edition (CELF-5) (Wiig et al., 2013) and the Comprehensive Assessment of Spoken Language, Second Edition (CASL-2) (Carrow-Woolfolk, 2017) do include age norm-references up to the age of 21;11 (years;months), the participant samples from which the norms are drawn are either extrapolated from younger clinical samples (as is the case with the CELF-5) or include participants with other clinical diagnoses, such as autism (as is the case with the CASL-2) (Choi-Tucci et al., 2024). Therefore, we opted to utilize the TILLS for the current study, with all standard scores normed to 18 years of age for all participants to derive the five TILLS composite scores (sound/word, sentence/discourse, oral, written, and identification core score). Individual composite score profiles for each participant with DLD, divided into quartiles, and the distribution of raw scores across each TILLS subtest are presented in Supporting Information Figures S1 and S2, respectively (Supporting Information can be found at https://doi.org/10.1162/NOL.a.277). Participants also completed the Block Design and Matrix Reasoning subtests of the Wechsler Abbreviated Scale of Intelligence–Second Edition (Wechsler, 2018) and had to have a *T* score above 30 (equivalent to a standard score of 70) on at least one of the subtests to be included in the study (Table 1). One participant with DLD did not complete the Matrix Reasoning subtest due to examiner error but scored above 30 on the Block Design subtest and was therefore retained in the sample.

Group differences across the five TILLS composite measures were evaluated using a multivariate analysis of variance (MANOVA). The dependent variables included each of the composite scores and group (DLD vs. TD) served as the between-subjects factor. Follow-up univariate ANOVAs were conducted for each assessment to identify which measures contributed to the overall group difference. Partial eta-squared (*η*^2^) was calculated as an effect size for each ANOVA. All tests used an alpha level of 0.05.

### MRI Data Acquisition

#### Procedure

Imaging data were acquired on a 3T Siemens Prisma scanner with a 64-channel head coil at the University of Washington Center for Human Neuroscience. Participants were fitted with earplugs and noise-canceling headphones (Optoacoustics), and foam padding was added around participants’ heads to minimize head movement. High-resolution structural images were acquired using a T1-weighted, rapid gradient echo sequence and parallel imaging (GRAPPA) with an acceleration factor of 2. The parameters were as follows: repetition time (TR) = 2,500 ms, echo time (TE) = 1.8 ms, inversion time = 1,000 ms, flip angle = 8°, 0.8 mm^3^ slice thickness, acquisition matrix = 320 × 300, scan time = 8 min 9 s.

Whole-brain functional BOLD data were acquired using a multiband gradient echo planar imaging (EPI) sequence with the following parameters: TR = 2,000 ms, TE = 30 ms, flip angle = 79°, multiband acceleration factor = 4, slice thickness = 179 slices at 2 mm^3^, matrix size = 106 × 106, field of view (FOV) = 212 × 212 mm^2^, 80 phase encoding steps with an anterior-to-posterior phase encoding direction, scan time = 6 min 10 s. To correct for susceptibility-induced geometric distortions in the functional MRI data, a pair of spin–EPI fieldmaps with opposite phase-encoding directions (anterior to posterior [AP] and posterior to anterior [PA]) were acquired prior to functional imaging. Each fieldmap consisted of 76 interleaved transverse slices aligned to the AC–PC plane, with an FOV of 212 × 212 mm, resulting in 2.0 mm^3^ voxels. Sequence parameters were as follows: TR = 2,000 ms, TE = 30 ms, flip angle = 79°, and echo spacing = 0.66 ms, with a multiband acceleration factor of 4.

#### Language localizer task

To functionally identify language-responsive regions in individual participants, we used the well-validated language localizer task developed by Scott et al. (2017). All materials for the language localizer task can be downloaded here: https://www.evlab.mit.edu/resources-all/download-localizer-tasks. This blocked design task contrasts brain responses to auditorily presented natural sentences (intact speech condition) with responses to acoustically degraded (degraded speech condition) versions of the speech stimuli. Importantly, this task was designed to reduce cognitive demands on the listener, as it is considered a passive, attention-free listening task that is still engaging enough for the listener to prevent mind-wandering.

Before scanning, participants were informed that they would hear a mix of intelligible and unintelligible audio clips and were instructed to keep listening, even if they did not understand the speaker. To ensure that the volume level was set to an appropriate loudness, we played a recording of the “Rainbow Passage” (Fairbanks, 1960) during the fieldmap sequences. The passage contains all the sounds of American English and was adjusted to match the amplitude of the intact stimuli (Sevitz et al., 2021). This ensured that, coupled with the noise-canceling headphones, participants could comfortably hear the auditory stimuli over the scanner sounds. During the fMRI task, stimuli were presented auditorily through MATLAB (MathWorks) while participants watched a fixation point on the screen. During silent conditions (baseline), participants saw a black fixation cross on a white background, and during both speech conditions (intact and degraded), participants saw a black circle on a white background.

As described in Scott et al. (2017), each block consisted of a single audio clip (intact or degraded speech), followed by a fixation period (silent condition). The intact speech stimuli consisted of sentences sourced from the Moth podcast, TED Talks, and celebrity interviews. The degraded speech stimuli were created by copying the intact speech stimuli and low-pass filtering them with a modulated noise overlay that preserved the amplitude envelope of the original speech but disrupted intelligibility. The resulting degraded clips resembled muffled or poor radio reception and were unintelligible to listeners; however, unlike reversed speech—which is frequently used in language localizer paradigms—the clips still sounded like naturalistic speech. Full technical details of the degradation procedure are available in Scott et al. (2017).

The original stimuli from Scott et al. (2017) were pseudorandomly ordered and balanced across conditions, such that each participant in the current study heard the same stimuli. In the original Scott et al. (2017) paper, participants underwent two functional runs; however, J. J. Lee et al. (2024) demonstrated that the functional language network can be reliably localized within 3.5 min of scanning (J. J. Lee et al., 2024). Therefore, we only included the first functional run to optimize scan time. The run contained 16 experimental blocks (eight intact, eight degraded) and five fixation blocks. The duration of the experimental blocks was 18 s, while the duration of the fixation period was 14 s, for a total of 358 s (∼6 min).

### fMRI Data Preprocessing

The functional data were preprocessed and analyzed using FEAT (fMRI Expert Analysis Tool, Version 6.00) as part of the FSL package (FMRIB Software Library, Version 6.0.7.4, www.fmrib.ox.ac.uk/fsl). Preprocessing steps included motion correction using MCFLIRT (Jenkinson et al., 2002), followed by brain extraction using BET (Smith, 2002) to remove non-brain tissue. Spatial smoothing was applied with a Gaussian kernel of 5 mm FWHM, and high-pass temporal filtering was used to remove low-frequency signal drifts with a cutoff of 100 s. Fieldmap-based EPI distortion correction was applied using magnitude and phase difference images with an effective echo spacing of 0.16499875 ms (due to the acceleration factor of 4) and a total readout time of 30 ms, with a posterior-to-anterior phase encoding direction (*y*−). Note, for one participant, the magnitude and phase images were not generated due to operator error. Thus, to correct for fieldmap distortions for this participant, we created the magnitude and phase difference images using FSL’s *topup* tool and then applied the same parameters listed above to these generated magnitude and phase difference images (Andersson et al., 2003). Registration of functional images to each subject’s high-resolution T1-weighted structural scan was performed using boundary-based registration (Greve & Fischl, 2009), and the aligned images were then nonlinearly registered to MNI152 standard space using FNIRT (12 *df*) (Andersson et al., 2019).

Individual activation maps were derived with a general linear model using FSL’s FILM (FMRIB’s Improved Linear Model) time-series modeling tool. The design matrix included two explanatory variables: intact speech and degraded speech. Events were modeled as blocks with durations corresponding to the length of each stimulus presentation (e.g., 18 s) and were convolved with FSL’s canonical gamma hemodynamic response function. Temporal derivatives of explanatory variables were included. Custom timing files specified the onset and duration of each event. Null events (i.e., silence between conditions) were not modeled.

Three contrasts were defined at the first level: (1) intact, which provides information about both higher-level language activation and lower-level auditory brain activation; (2) degraded, which provides information about lower-level auditory activation to speech without linguistic content; and (3) Intact > Degraded, which isolates activation for higher-level language processes. The resulting contrast parameter estimates for each participant were carried forward to group-level analyses.

### Group-Level Analyses

Group-level (between-subjects) analyses were performed using FSL’s FEAT. To assess group-level effects for the intact speech contrast, degraded speech contrast, and Intact > Degraded speech contrast, we employed nonparametric permutation testing using FSL’s *randomise* tool (Woolrich et al., 2004). *Randomise* was run with 5,000 permutations to build the null distribution of the test statistic, allowing for robust, assumption-free inference. *Z*-statistic images were thresholded nonparametrically using clusters determined by *z* > 3.1 and a (corrected) cluster significance threshold of *p* = 0.05.

At the group level, analyses were conducted using a general linear model (GLM). Group membership was modeled as a between-subjects factor. To account for potential confounding effects of head motion, the mean relative displacement from the first-level analysis was included as a covariate. Four statistical tests were evaluated for each task contrast: (1) TD group activation, (2) DLD group activation, (3) TD > DLD, and (4) DLD > TD. These contrasts allowed us to examine average activation patterns for each group across the whole brain, as well as relative differences between groups.

### Overlap Analyses

To better understand within-group and between-groups differences in activation patterns for the Intact > Degraded comparison, we generated overlap maps from individual activation results (*t*-statistic maps). Each map was thresholded at *t* > 2.3 and binarized to indicate suprathreshold activation. The resulting binary activation masks were then summed within each group (TD or DLD) to create group-level overlap maps. In the overlap images, each voxel intensity represents the number of participants within a group showing suprathreshold activation at that voxel. These maps were visualized on the MNI152 template to qualitatively assess the spatial consistency and extent of activation within and between groups.

### Relationship With Behavior

In addition to the group comparisons, we conducted separate regression analyses to examine the relationship between brain activation and language performance: (1) across all participants, (2) within the DLD group, and (3) within the TD group. For these analyses, individual standard scores on the TILLS Sentence and Discourse Composite were included as the predictor of interest to capture higher-level language processing. A single contrast was specified to test regions in which activation to the Intact > Degraded contrast was associated with higher TILLS performance. As the Intact > Degraded condition isolates activation for linguistic information specifically, we expected to find that better scores on the TILLS Sentence and Discourse Composite would be related to activation within left-hemisphere frontotemporal language regions (Scott et al., 2017).

## RESULTS

### Behavioral Results

Participant scores on the online Fidler protocol are shown in Figure 1. As previously mentioned, one participant with a history of DLD scored within the TD range; however, we opted to include them in this sample as they have a childhood diagnosis of DLD from a licensed SLP. Results are identical if this participant is instead excluded from all analyses.

**Figure 2.**
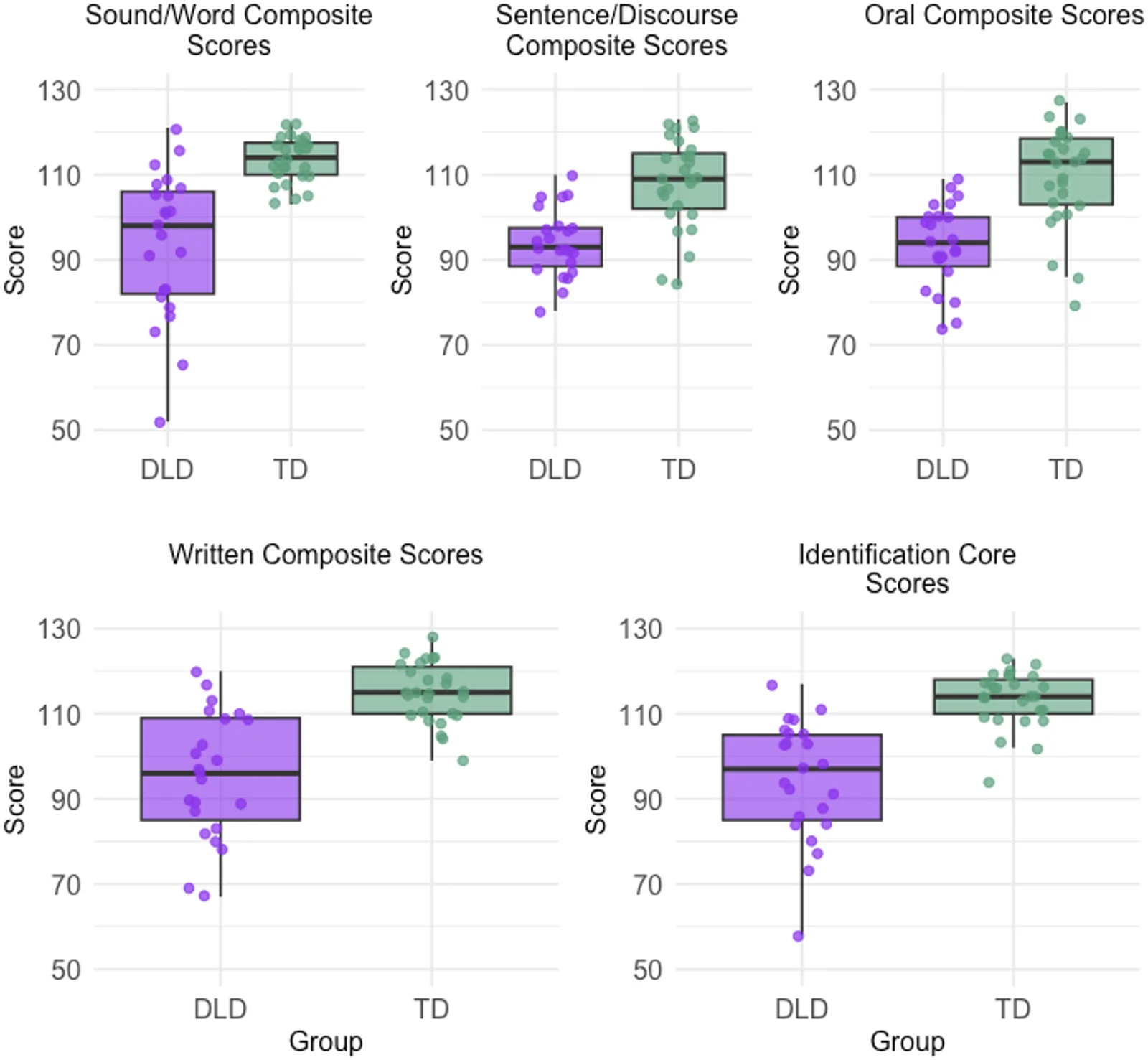
Test of Integrated Language and Literacy Skills (TILLS) composite and identification core scores for adults with developmental language disorder (DLD) and adults with typical language development (TD). Differences between groups were significant for all TILLS composite scores and the identification core scores at *p* < 0.001 (based on univariate ANOVAs).

Standard scores for the TILLS composites and identification core are shown in Figure 2. A one-way MANOVA revealed a significant multivariate effect of group on the combined assessment measures, Wilks’s Λ = 0.47, *F*(5, 44) = 9.85, *p* < 0.001. Follow-up univariate ANOVAs revealed significant group differences on all five TILLS composite measures. Large effects were observed for the sound/word composite, *F*(1, 48) = 32.57, *p* < 0.001, partial *η*^2^ = 0.40; the sentence/discourse composite, *F*(1, 48) = 26.91, *p* < 0.001, partial *η*^2^ = 0.36; the oral composite, *F*(1, 48) = 27.17, *p* < 0.001, partial *η*^2^ = 0.36; the written composite, *F*(1, 48) = 37.21, *p* < 0.001, partial *η*^2^ = 0.44; and the identification core score, *F*(1, 48) = 38.65, *p* < 0.001, partial *η*^2^ = 0.45. Overall, the DLD group scored significantly lower than the TD group across all domains with large effect sizes for each assessment. However, based on the TILLS Identification Core score, only eight individuals in our sample met diagnostic criteria for DLD. This represents approximately 35% of the participants identified as having DLD by the Fidler protocol. These results parallel the findings reported by Choi-Tucci et al. (2024), who found that 36% of participants identified as having DLD by the Fidler protocol also met diagnostic criteria on the TILLS, suggesting that the TILLS may not fully capture the difficulties faced by adults with DLD.

### Neuroimaging Results: Higher-Level Language Processing

We compared the fMRI BOLD responses to a passive language localizer task between adults with and without DLD. Results were examined to explore group differences in activation across the whole brain to (1) intact speech, (2) degraded speech, and (3) Intact > Degraded speech. The canonical language network is found in the Intact > Degraded contrast, as it removes activation related to lower-level auditory processing, thereby isolating neural activity associated with higher-level language processing. Below, we start by presenting results for the degraded and intact conditions and then focus on the comparison of interest, Intact > Degraded speech.

### Degraded Speech Condition

**Figure 3.**
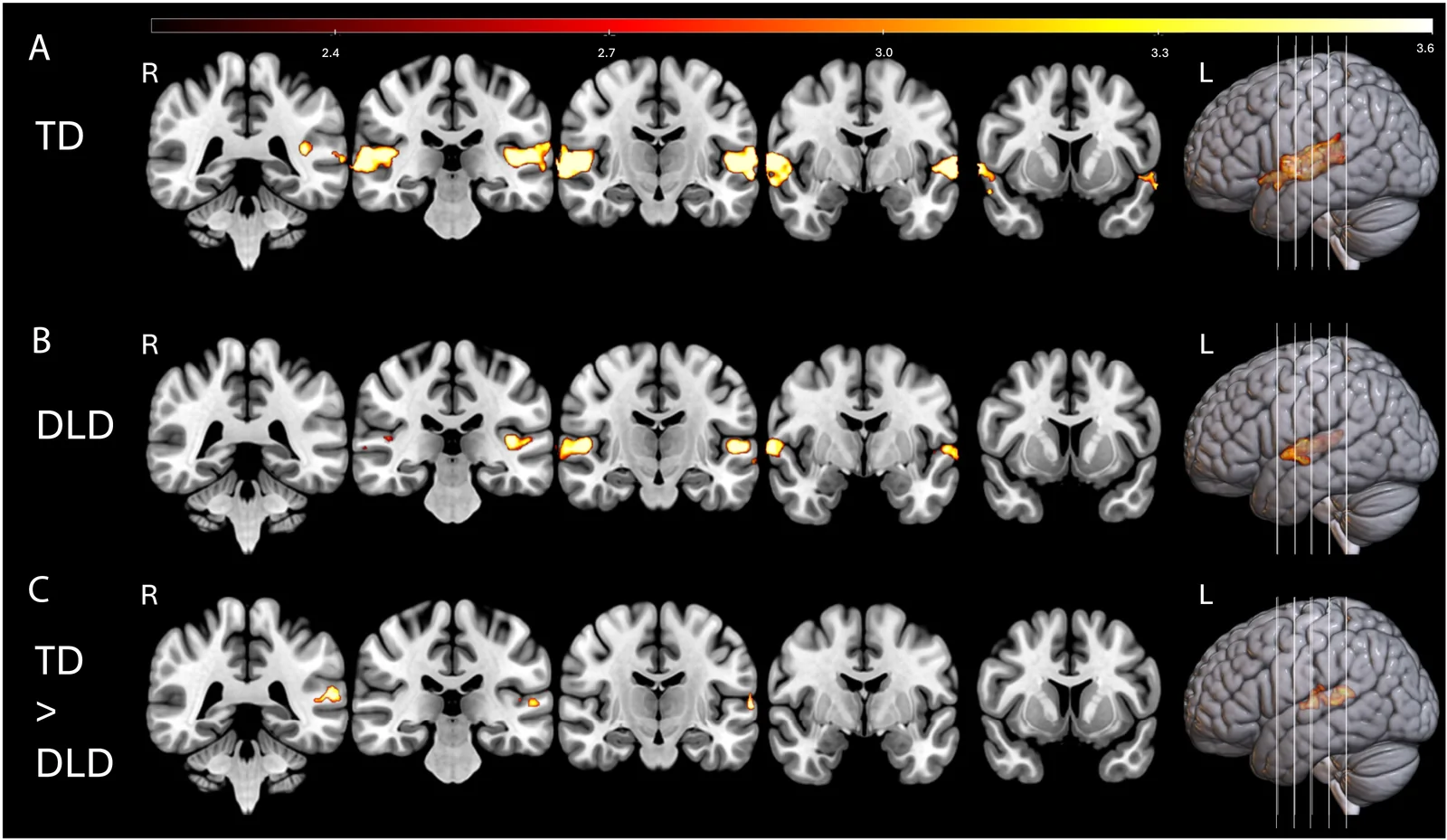
Group average BOLD activation patterns during the degraded speech condition for (A) adults with typical language development (TD), (B) adults with developmental language disorder (DLD), and (C) regions showing greater activation for the TD group compared to the DLD group (TD > DLD). Results are displayed at a cluster threshold of *Z* > 3.1, *p* < 0.05 (cluster-corrected), on the MNI-152 1-mm template brain. The color bar reflects voxelwise *z* statistics within significant clusters. The right hemisphere (R) is displayed on the left side of the image for the coronal slices, while the 3D brain image shows the left hemisphere (L).

Significant regions of activation for the TD group in the degraded speech condition included large clusters of activation in the left (15,539 voxels, *p* = 0.0012) and right (16,401 voxels, *p* = 0.0012) hemispheres (see Supporting Information Table S1 for peak coordinates and statistics), including bilateral planum temporale (PT), supramarginal gyrus (SMG), Heschl’s gyri (HG), and superior temporal gyrus (STG) with extension into surrounding regions including the middle temporal gyrus (MTG), planum polare (PP), postcentral gyri (PCG), and central opercular cortex (COP) (Figure 3A). The DLD group showed similar regions of activation in the right (5,617 voxels, *p* = 0.0032) and left (5,341 voxels, *p* = 0.0034) hemispheres, with the exception of the SMG, which was not significant for the DLD group (Figure 3B). Results for the TD > DLD contrast (Figure 3C) revealed that activation (2,722 voxels, *p* = 0.036) in the left PT, SMG, STG, PCG, and COP was weaker in the DLD group compared to the TD group. Taken together, these findings indicate broadly similar recruitment of auditory language regions across groups, with the TD group showing stronger activation in left-hemisphere areas associated with phonological and sensorimotor processing. No regions showed greater activation for the DLD group relative to the TD group (DLD > TD).

### Intact Speech Condition

**Figure 4.**
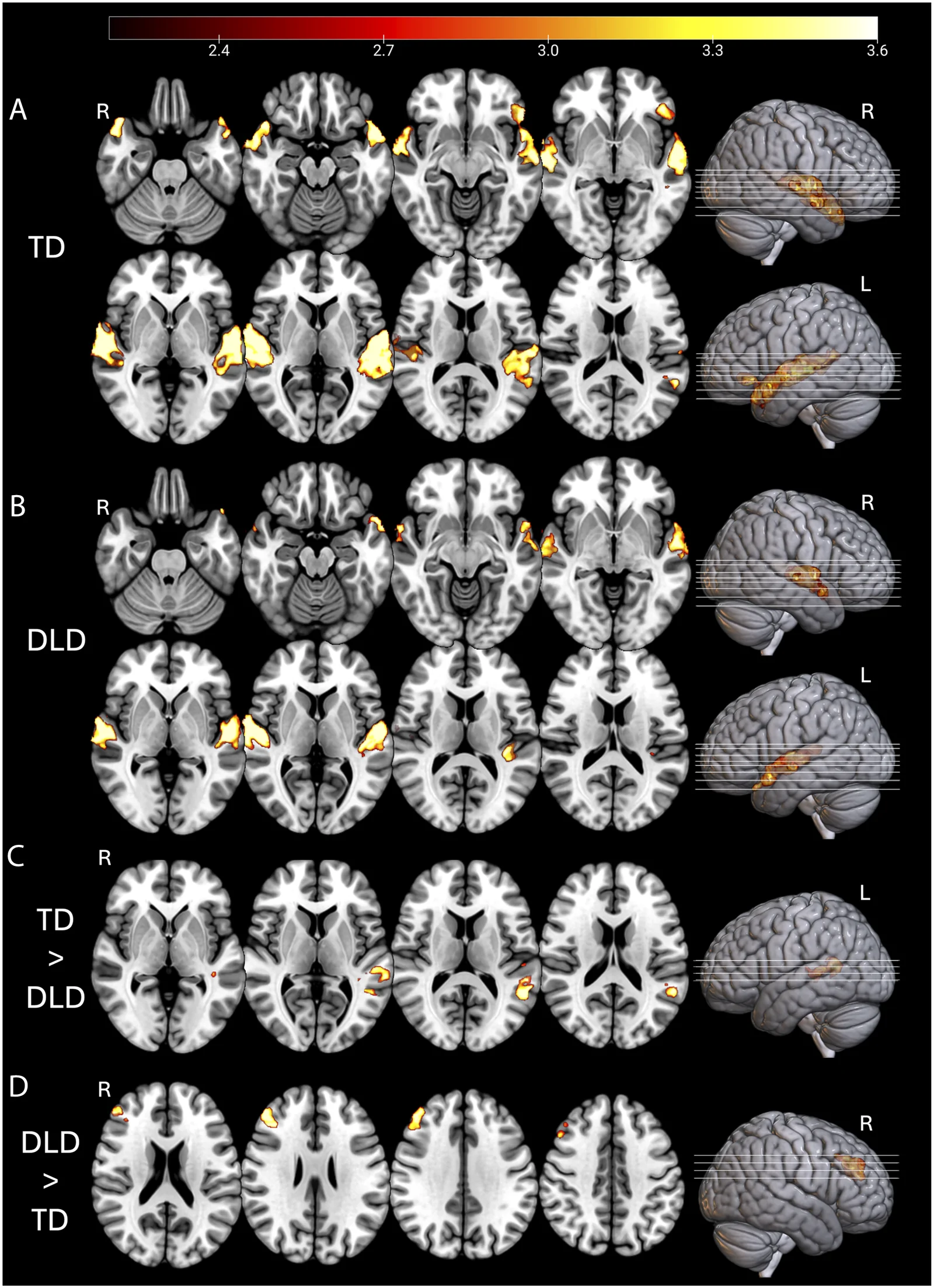
Group average BOLD activation patterns during the intact speech condition for (A) adults with typical language development (TD), (B) adults with developmental language disorder (DLD), (C) regions showing greater activation for the TD group compared to the DLD group (TD > DLD), and (D) regions showing greater activation for the DLD group compared to the TD group (DLD > TD). Results are displayed at a cluster threshold of *Z* > 3.1, *p* < 0.05 (cluster-corrected), on the MNI-152 1-mm template brain. The color bar reflects voxelwise *z* statistics within significant clusters. The right hemisphere (R) is displayed on the left side of the image. The 3D sagittal brain image shows the right (top) and left (L; bottom) hemispheres for the TD and DLD groups. For the group comparisons (C and D), the 3D sagittal image shows the L and R hemispheres, respectively.

Results for the TD group in the intact speech condition revealed two large clusters of activation in the left (26,823 voxels, *p* = 0.001) and right (18,924 voxels, *p* = 0.0014) hemispheres (see Supporting Information Table S2 for peak coordinates and statistics), consistent with Scott et al. (2017). Adults with TD had widespread activation in bilateral auditory and language comprehension regions including the PT, MTG, STG, temporal pole (TP), PP, HG, SMG, COP/parietal opercular cortex, left angular gyrus (AG), the pars triangularis portion of the left IFG, and the right PCG (Figure 4A). The DLD group activated similar auditory-phonological brain regions in the left (9,668 voxels, *p* = 0.0008) and right (7,200 voxels, *p* = 0.0014) hemispheres (Figure 4B) but had reduced activation relative to the TD group (TD > DLD) in a cluster of voxels (3,406 voxels, *p* = 0.0232) in the left AG that extended into the STG, PT, and SMG (Figure 4C). This pattern suggests more robust engagement of left-lateralized auditory and language networks in the TD group during intact speech comprehension. Conversely, the DLD > TD contrast revealed greater activation for the DLD group in a cluster of voxels (3,541 voxels, *p* = 0.021) in the right frontal pole extending into the middle frontal gyrus (Figure 4D). In summary, adults with DLD demonstrated weaker activation in left-hemisphere speech comprehension regions and increased recruitment of right frontal domain-general areas, indicating a different pattern of neural engagement during intact speech processing compared to adults with TD.

### Intact > Degraded Speech Condition

**Figure 5.**
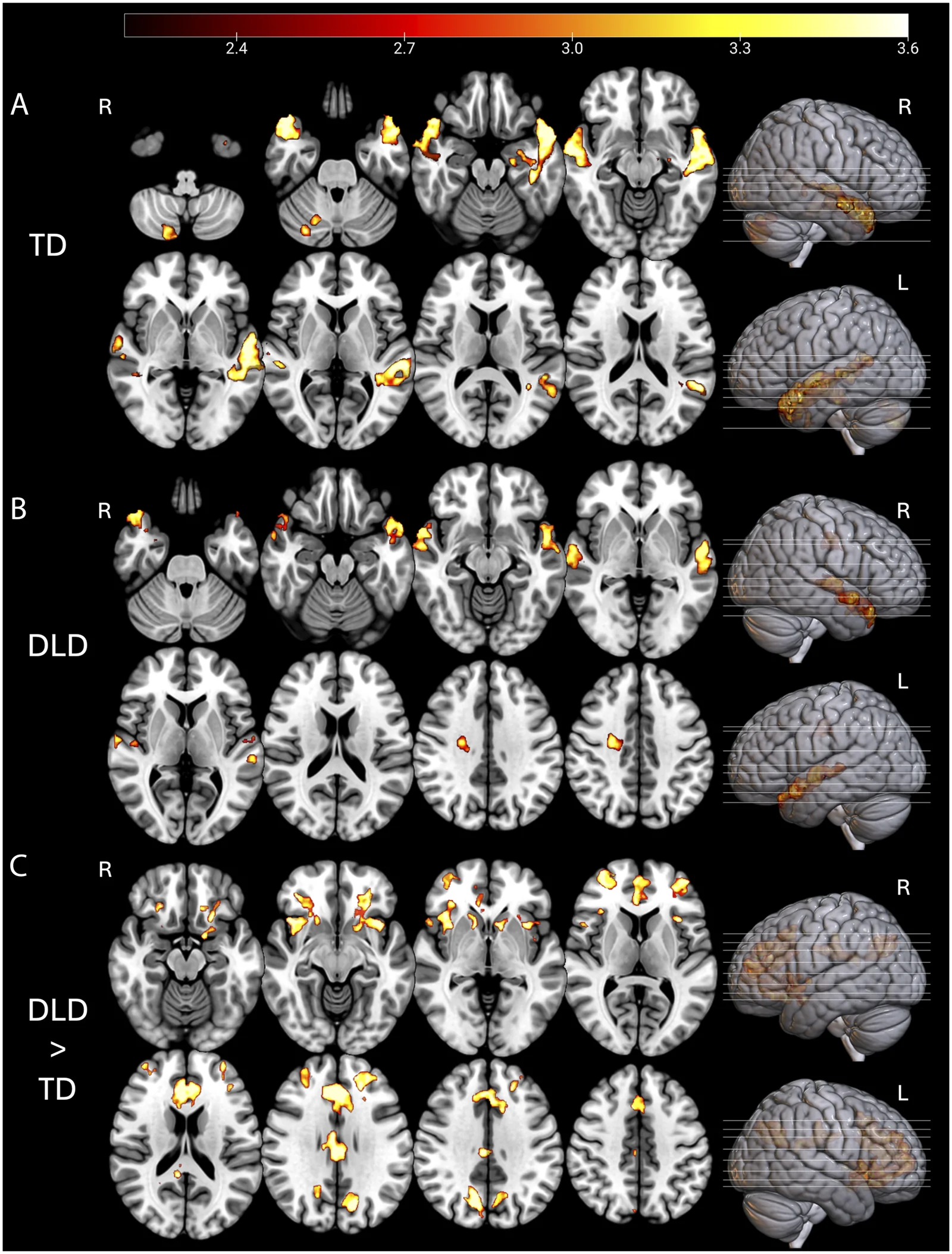
Group average BOLD activation patterns during the Intact > Degraded speech condition for (A) adults with typical language development (TD), (B) adults with developmental language disorder (DLD), and (C) regions showing greater activation for the DLD group compared to the TD group (DLD > TD). Results are displayed at a cluster threshold of *Z* > 3.1, *p* < 0.05 (cluster-corrected), on the MNI-152 1-mm template brain. The color bar reflects voxelwise *z* statistics within significant clusters. The right hemisphere (R) is displayed on the left side of the image. The 3D sagittal brain image shows the right (top) and left (L; bottom) hemispheres.

For the TD group, the Intact > Degraded contrast revealed widespread bilateral activation across core language regions, including the STG, extending into temporoparietal areas (Figure 5A). The largest cluster (29,842 voxels, *p* = 0.001) included the left STG, MTG, superior temporal sulcus (STS), PT, TP, inferior temporal gyrus (ITG), SMG, and AG (see Supporting Information Table S3 for peak coordinates and statistics). A second cluster (14,188 voxels; *p* = 0.0026) encompassed the right TP extending into the STG, MTG, HG, and PP, reflecting bilateral recruitment of temporal regions involved in intelligible speech comprehension. A smaller cluster (6,025 voxels; *p* = 0.0136) was observed in the right cerebellum, including Crus I/II and lobules VI and VIIb, which may reflect semantic and syntactic processing as well as domain-general attention mechanisms (Li et al., 2020; Nakatani et al., 2023; Stoodley et al., 2012). Importantly, these findings, including right cerebellar involvement in higher-level linguistic processing, align with the findings from the original language localizer study from Fedorenko et al. (2010). Overall, the results from this study confirm that adults with TD recruit a broad, bilateral network associated with the comprehension of meaningful speech, beyond lower-level auditory processing.

In the DLD group, the Intact > Degraded contrast also revealed widespread bilateral activation across temporal and frontal lobe regions, though clusters were somewhat smaller in extent than those observed in the TD group (Figure 5B). The largest cluster (7,308 voxels; *p* = 0.004) was centered in the left STG and MTG, including portions of the STS, PT, and PP, consistent with engagement of core left-hemisphere language areas. A second cluster (7,237 voxels; *p* = 0.004) encompassed the right TP and portions of the STG and MTG reflecting bilateral recruitment of temporal regions supporting higher-level linguistic processing. A smaller cluster (2,055 voxels; *p* = 0.0346) was observed in the right superior frontal gyrus, including the premotor and medial prefrontal cortex, likely reflecting domain-general attentional or executive processes supporting speech comprehension. Overall, the DLD group showed a broadly similar bilateral pattern of higher-level language network activation to the TD group, though clusters were generally smaller, suggesting somewhat reduced or less extensive recruitment of these regions.

A direct group comparison for the Intact > Degraded speech contrast revealed no regions of significantly greater activation for the TD group relative to the DLD group (TD > DLD). In contrast, the reverse comparison (DLD > TD) revealed multiple cluster regions where the DLD group had significantly more activation than the TD group (Figure 5C). Greater activation was observed bilaterally in the anterior cingulate cortex (ACC), posterior cingulate cortex (PCC), the right frontal pole with extension into the frontal orbital cortex and the middle frontal gyrus, the left superior frontal gyrus with extension into the frontal and middle gyri, the bilateral insular cortices, and the bilateral structures of the basal ganglia, including precuneus, caudate nucleus, and putamen. This pattern of results indicates increased engagement of midline and fronto-insular regions, as well as subcortical structures, which suggests that adults with DLD may recruit additional networks associated with domain-general control and attention during higher-level language processing.

### Between-Groups and Within-Group Activation Overlap

**Figure 6.**
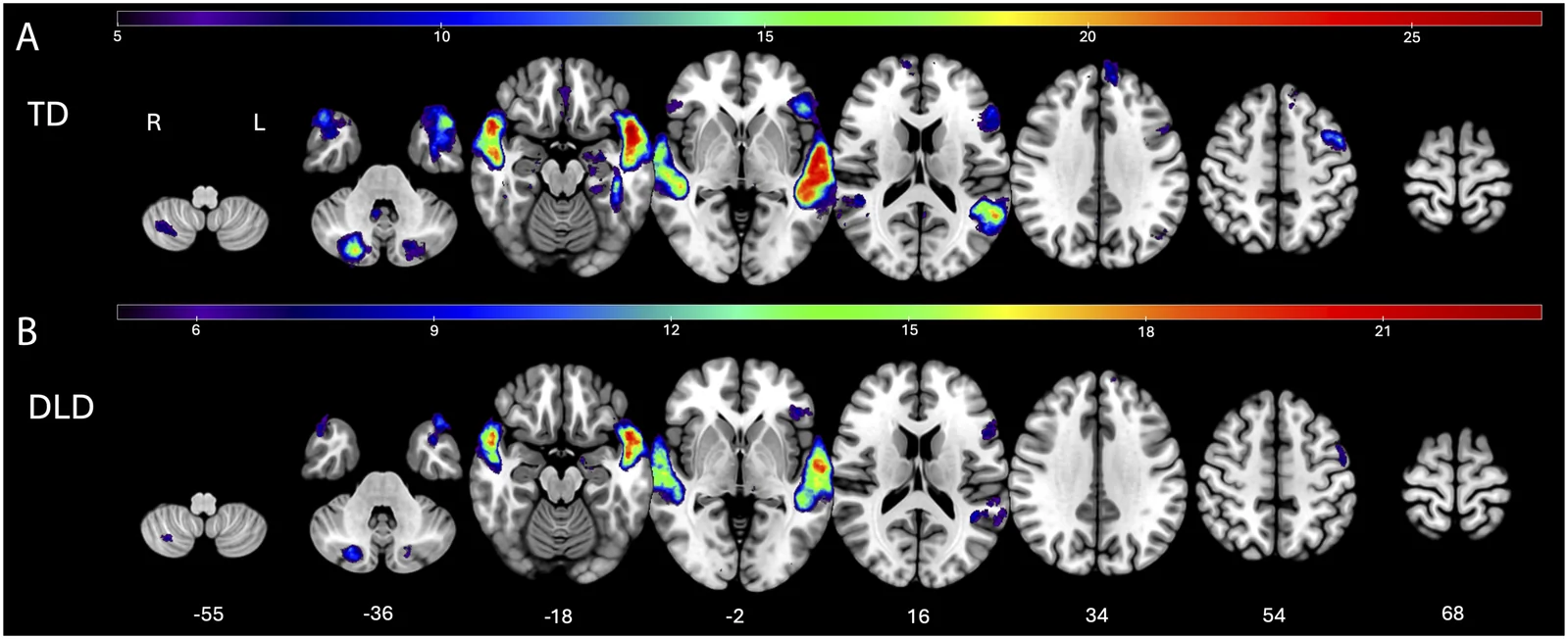
Activation overlay map for the Intact > Degraded contrast for (A) adults with typical language development (TD; *n* = 27) and (B) adults with developmental language disorder (DLD; *n* = 23). The color bar indicates the number of participants within each group with suprathreshold activation (*t* > 2.3, *p* < 0.05) at that voxel. Note that the scale of the color bar begins at 5 and differs in the total number of participants between groups. Activation patterns are displayed on the MNI-152 1-mm template brain. The right hemisphere (R) is displayed on the left side of each brain slice. Axial slice labels are provided at the bottom of the image.

An activation overlap map was created to visually inspect differences between and within groups for the Intact > Degraded contrast. Visual inspection of the maps revealed clear differences in the consistency and spatial extent of activation between groups (Figure 6). The TD group showed broader and more consistent activation across frontal and temporal regions, particularly within the left IFG and superior temporal cortex. Nearly half of the participants with TD demonstrated suprathreshold activation in the left IFG. The TD group also demonstrated high levels of overlap extending posteriorly into the STG and MTG.

In contrast, the DLD group exhibited less within-group overlap, indicating greater variability in activation patterns. Visually, activation in the left IFG was sparse, with only a few participants with DLD showing suprathreshold voxels in this region. Similarly, overlap in the temporal lobe, particularly the STG and MTG, was markedly reduced relative to the TD group. These patterns suggest that adults with DLD recruited the canonical frontotemporal language network but did so less consistently and to a lesser extent than adults with TD during the language localizer task.

### Relationship Between Activation Patterns and Language Abilities

**Figure 7.**
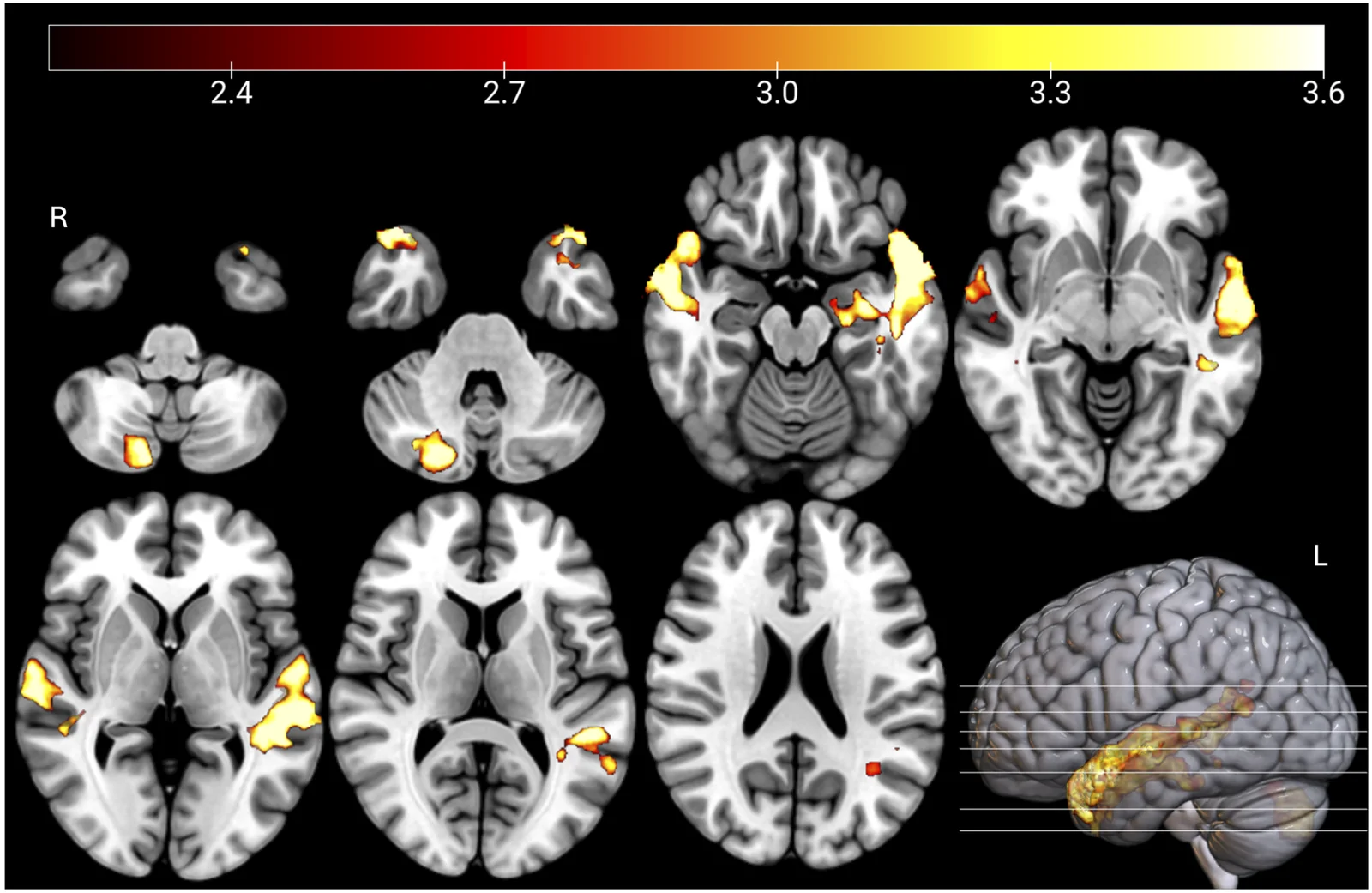
Brain regions showing significant positive correlations between activation for the Intact > Degraded speech condition and scores on the Test of Integrated Language and Literacy Skills (TILLS) Sentence/Discourse Composite. Scores were modeled as a continuous variable across all participants. Results are displayed at a cluster threshold of *Z* > 3.1, *p* < 0.05 (cluster-corrected), on the MNI-152 1-mm template brain. The color bar reflects voxelwise *z* statistics within significant clusters. The right hemisphere (R) is displayed on the left side of the image. The 3D sagittal brain image displays the left hemisphere (L).

To examine how individual differences in higher-level language abilities relate to brain activation during speech comprehension, a voxelwise GLM was conducted using participants’ TILLS Sentence and Discourse Composite scores as a covariate for the Intact > Degraded contrast (Figure 7). Models were run across the entire cohort, within just the DLD group, and within just the TD group.

#### Across entire cohort

Higher composite scores, reflecting stronger sentence- and discourse-level processing skills, were associated with greater activation in a large left-hemisphere cluster encompassing the STG and extending into the MTG, STS, AG, SMG, and inferior and anterior temporal cortex (29,332 voxels, *p* = 0.0008) (see Supporting Information Table S4 for peak coordinates and statistics). A corresponding cluster in the right superior TP extending into the MTG, STS, HG, and PT (15,509 voxels, *p* = 0.0026) also showed a positive relationship with TILLS performance (see Supporting Information Table S4 for significant cluster coordinates). Additional activation was observed in the right cerebellar Crus I/II, VI, and VIIb (5,426 voxels, *p* = 0.019). Collectively, these findings suggest that, across groups, individuals with stronger sentence- and discourse-level language skills recruit left-hemisphere (and, to a lesser extent, right-hemisphere) temporal regions, as well as cerebellar regions associated with language, more robustly during higher-level speech processing, consistent with models of language processing (Fedorenko et al., 2011; Malik-Moraleda et al., 2022).

#### Within DLD group

**Figure 8.**
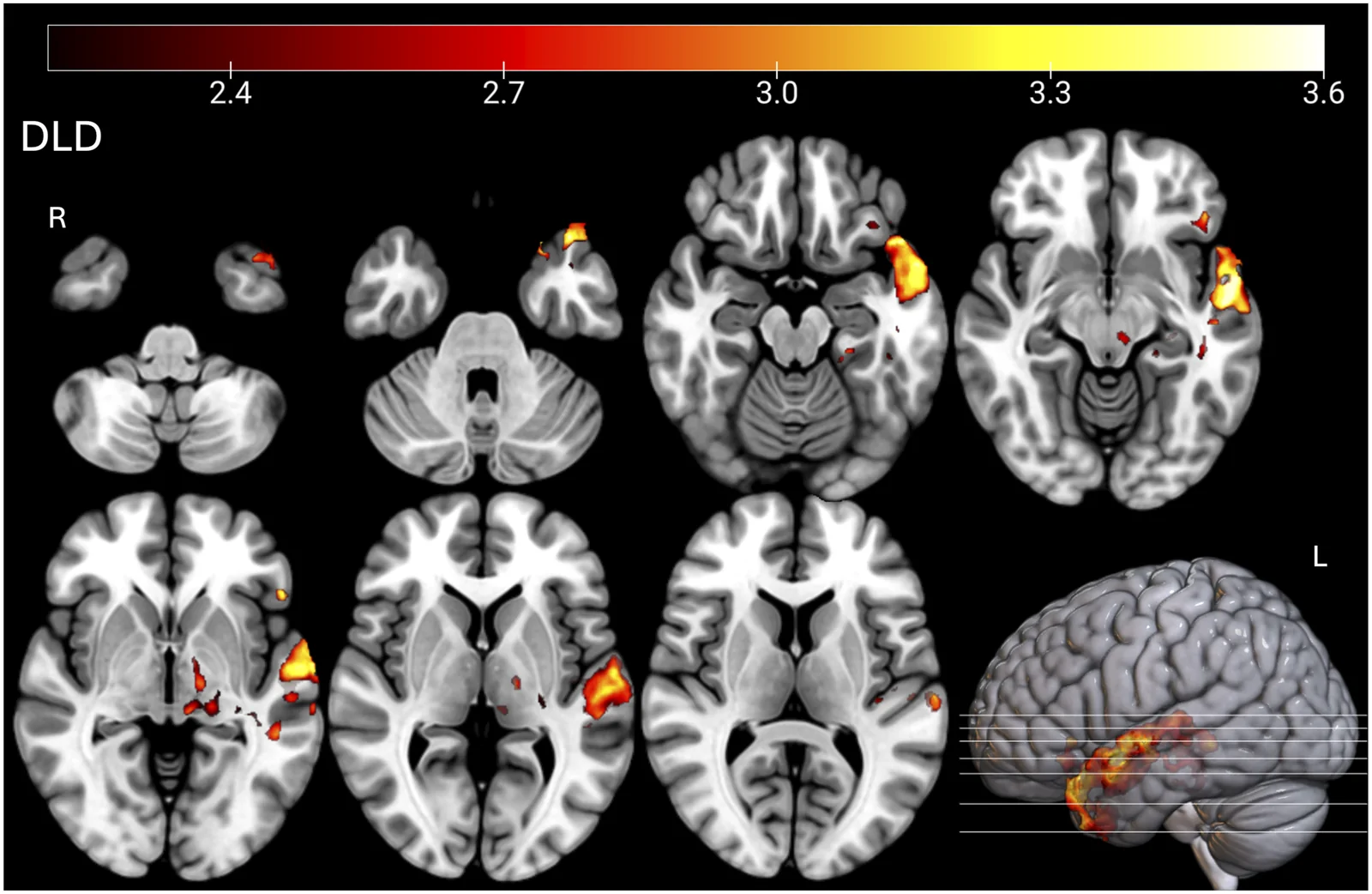
Brain regions showing significant positive correlations in the DLD group only between activation for the Intact > Degraded speech condition and scores on the Test of Integrated Language and Literacy Skills (TILLS) Sentence/Discourse Composite. Results are displayed at a cluster threshold of *Z* > 3.1, *p* < 0.05 (cluster-corrected), on the MNI-152 1-mm template brain. The color bar reflects voxelwise *z*-statistics within significant clusters. The right hemisphere (R) is displayed on the left side of the image. The 3D sagittal brain image displays the left hemisphere (L).

Within the DLD group, higher TILLS Sentence and Discourse Composite scores were associated with greater activation in a single large left-hemisphere cluster (22,653 voxels, *p* = 0.0206) encompassing the STG extending into the MTG, TP, PT, HG, PP, IFG, frontal operculum, thalamus, hippocampus, and putamen (Figure 8; see Supporting Information Table S4 for significant cluster coordinates). This pattern suggests that within the DLD group, stronger sentence- and discourse-level language abilities are associated with broader recruitment of canonical language regions as well as subcortical structures, indicating that individual differences in language outcomes may be partially explained by the degree to which adults with DLD engage typical frontotemporal language circuitry. The emergence of subcortical regions including the thalamus, hippocampus, and putamen is noteworthy and may reflect additional demands on corticosubcortical coordination and memory systems in this group, though these findings should be interpreted cautiously given the exploratory nature of these analyses and the reduced sample size within the DLD group alone.

#### Within TD group

No significant clusters were identified in the TD group, suggesting that the language–brain relationship observed in the full sample analysis is largely driven by variability within the DLD group.

## DISCUSSION

The present study examined the neural basis of language processing in adults with and without DLD using an fMRI paradigm designed to isolate higher-level linguistic processing from lower-level auditory perception. Participants passively listened to intelligible (intact) and acoustically degraded speech while in the MRI scanner, allowing comparison between patterns of brain activation across conditions and groups. Given the relatively robust language skills in this cohort and the simplicity of the task, we hypothesized a priori that activation patterns within canonical language networks would be largely similar between groups, while better performance on the TILLS Sentence and Discourse Composite would be related to activation within canonical frontotemporal language regions.

### Degraded Speech Processing

Overall, both groups showed similar activation patterns during the degraded speech condition, though the DLD group had several areas of reduced activation relative to the TD group. Nonetheless, the results suggest that both participants with TD and DLD recruit a broadly similar auditory network when listening to degraded speech, including key regions involved in early auditory processing (e.g., PT, HG, and STG) (Fedorenko et al., 2024; Norman-Haignere et al., 2015) and higher-order integration (MTG and adjacent areas) (Hickok & Poeppel, 2007).

Although this condition was designed to minimize access to lexical, semantic, and syntactic content, both groups showed reliable activation of the MTG. This pattern is consistent with Scott et al. (2017), from which our stimuli and paradigm were adapted, and likely reflects the strong sensitivity of left temporal regions to speech intelligibility even when intelligibility is severely reduced (Erb et al., 2013; Obleser & Kotz, 2010). Importantly, unlike paradigms that use isolated nonwords or noise-vocoded stimuli, our approach employed clips of real talks and podcasts that were acoustically degraded to remove linguistic content but preserve the natural prosodic and temporal structure of speech (Scott et al., 2017). Prior work suggests that such naturalistic cues can encourage listeners to attempt comprehension even when meaning is unavailable (Bautista & Wilson, 2016), which may explain why MTG activity persisted despite the absence of usable linguistic information. For individuals with DLD, this demand to “search for meaning” under degraded conditions may be particularly challenging, potentially contributing to their reduced activation relative to adults with TD. It is worth noting that alternative control conditions such as reversed speech would not preserve these prosodic and temporal cues and thus may produce different activation patterns in lower-level speech processing regions. However, as Scott et al. (2017) argue, the choice of control condition is unlikely to substantially affect the identification of higher-level language regions, provided it lacks meaning and linguistic structure (a criterion met by the degraded speech used here).

Consistent with this interpretation, the TD group had more extensive engagement of the SMG, a region commonly associated with phonological working memory, sensorimotor integration, and the manipulation of speech sounds (Oberhuber et al., 2016). The absence of significant SMG activation in the DLD group may indicate reduced reliance on phonological or sensorimotor support mechanisms when speech perception is challenging. The group contrast (TD > DLD) further supports this interpretation, as those with DLD also showed weaker activation in left-hemisphere regions central to speech perception and phonological processing, including the PT, PCG, and COP (Grodzinsky & Amunts, 2006; Hickok & Poeppel, 2016; Liu et al., 2025; Oberhuber et al., 2016). Taken together, this pattern indicates that participants with DLD may engage left-lateralized auditory circuitry less efficiently than adults with TD, particularly under degraded listening conditions that place higher demands on speech (not language) processing.

### Intact Speech Processing

For the intact speech condition, both groups again engaged canonical auditory and language-related cortices. However, when directly compared, adults with DLD demonstrated less extensive activation within temporal and parietal regions (left STG, PT, SMG, and AG) and increased activation within right frontal areas, including the frontal pole and middle frontal gyrus. Contrary to our expectation of similar activation profiles, this pattern indicates that while adults with DLD recruit core language networks during intact speech comprehension, they do so to a lesser extent than their peers with TD, particularly in left temporal–parietal regions that support higher-order phonological processing and linguistic integration. Reduced activation in the left STG, PT, SMG, and AG aligns with longstanding behavioral evidence of weaknesses in phonological processing, verbal working memory, and linguistic integration (Estes et al., 2007; Gathercole & Baddeley, 1990; Leonard, 2014; Schwartz et al., 2013; Tallal & Piercy, 1974), along with neuroimaging studies that have reported atypical or reduced recruitment of left perisylvian language regions in individuals with DLD (Badcock et al., 2012; de Guibert et al., 2011; Hugdahl et al., 2004; Krishnan et al., 2021; Pigdon et al., 2020; Plante et al., 2017).

Notably, this reduced engagement of left temporal–parietal circuitry mirrors the pattern observed in the degraded speech condition, suggesting a more general tendency for individuals with DLD to under-recruit regions supporting phonological analysis and auditory–linguistic integration, regardless of whether speech is fully intelligible or perceptually challenging. Similarly, the emergence of right frontal recruitment during intact speech, including the frontal pole and middle frontal gyrus, may reflect increased reliance on domain-general executive or compensatory mechanisms to support comprehension. The frontal pole has been linked to higher-order executive processes such as goal-directed behavior, multitasking, and attentional control (Fassbender et al., 2006; Mansouri et al., 2017). The middle frontal gyrus contributes to reorienting attention (Thiel et al., 2004), working memory (Leung et al., 2002; Xu et al., 2024), and semantic priming (Rissman et al., 2003) and acts as a relay between the top-down dorsal and bottom-up ventral attention networks (Briggs et al., 2021). Although the specific functional role of the right frontal activity in this study cannot be determined from the current data, its presence is compatible with a compensatory account in which individuals with DLD engage additional neural resources while processing speech. Overall, the results from the degraded and intact speech conditions suggest a shift away from efficient, automatic processing toward more effortful top-down control processes.

### Higher-Level Linguistic Processing in Adults With DLD

For the Intact > Degraded contrast, adults with DLD showed greater activation than adults with TD primarily within bilateral midline and fronto-insular regions, including the ACC, PCC, insula, precuneus, and orbitofrontal cortex. These regions span components of the multiple-demand (also referred to as the central executive network), salience, and default mode networks, suggesting that adults with DLD may engage a broader constellation of domain-general control and integrative systems during higher-level linguistic processing.

### Large-Scale Brain Networks: Neural Compensation and Cognitive Costs

Broadly, the multiple-demand network is composed of regions in the inferior frontal sulcus, middle frontal gyrus, insular cortex, presupplementary/supplementary motor area, anterior/mid cingulate gyrus, and intraparietal sulcus that support goal-directed cognitive control (Fedorenko et al., 2013). The multiple-demand network is often co-activated during language comprehension when processing requires additional effort from extraneous task demands, but the network is not core to linguistic computations. Interestingly, in our participants with DLD, similar multiple-demand network regions were activated for the Intact > Degraded speech conditions, which may indicate that they exert greater cognitive effort to meaningfully interpret what they are hearing.

Unlike the multiple-demand network, the default mode network contributes to integrative and self-referential processing (Buckner et al., 2008; Raichle et al., 2001), while the salience network facilitates detection of and switching between salient external and internal stimuli, effectively coordinating engagement between the default mode network and the multiple-demand network (Goulden et al., 2014). This functional coupling among the default mode, multiple-demand, and salience networks typically increases across development (Uddin et al., 2011). In children with DLD, however, early disruptions to the language network may lead to an atypical reliance on these domain-general systems for linguistic processing, a reliance that may continue to strengthen into adulthood, but at the cost of reduced efficiency within the language network itself. Consistent with this possibility, recent work in children with DLD has shown widespread reductions in functional connectivity within the default mode network, cognitive control, and somatomotor networks (Doucet, Kruse, Eden, et al., 2025), indicating that atypical network organization may emerge early in development. These disruptions could interfere with the specialization or coordination of language networks, resulting in long-term differences in how language and cognitive control systems interact.

By adulthood, individuals with DLD often demonstrate age-appropriate linguistic performance but may rely on broader or less specialized neural pathways to achieve this as demonstrated in studies by Plante et al. (2006, 2017). One possibility is that while efficient language processing typically involves dynamic coordination between domain-general and domain-specific networks, adults with DLD may show less flexible segregation between these systems. Rather than selectively engaging frontotemporal language regions, they appear to recruit multiple large-scale networks simultaneously, a strategy that may support comprehension but at a higher cognitive cost. This is consistent with neural findings that the multiple-demand, default mode, and frontotemporal language networks support distinct computations in typically developing adults (Mineroff et al., 2018) and with behavioral findings that adults with DLD require more attention and effort for language processing (Hirn, 2023; Orrego et al., 2023). In fact, reduced efficiency in this functional segregation has been proposed as a hallmark of several other neurodevelopmental disorders. For example, individuals with dyslexia show reduced connectivity within frontoparietal control networks (Twait et al., 2018) and atypical recruitment of right-hemisphere or anterior regions during language tasks despite comparable behavioral performance (Kast et al., 2011; Richlan et al., 2011).

In the current study, adults with DLD engaged temporal regions associated with speech perception and comprehension, but they showed reduced recruitment of these regions as well as the IFG relative to adults with TD. This absence of robust frontal language activation, coupled with increased engagement of midline and insular regions, further supports the possibility that adults with DLD rely on additional domain-general systems to maintain attention and comprehension during the task. For instance, heightened activation in the anterior cingulate and insula, which are regions bridging the salience and multiple-demand networks, may reflect increased monitoring demands as participants sustain focus on the auditory input. Similarly, engagement of the posterior cingulate and precuneus, core hubs of the default mode network, could indicate compensatory recruitment of integrative or internally directed processes, such as drawing on contextual or semantic knowledge to support comprehension. Alternatively, this combination of activation may reflect less efficient modulation among large-scale systems, leading to concurrent activation of networks that are typically more segregated during well-practiced language processing. Taken together, these findings indicate that while adults with DLD can successfully process intelligible speech, they may do so through a broader and more effortful network configuration, particularly one that reflects compensatory recruitment and altered coordination among language-specific, control, and integrative systems.

### Subcortical Alterations and Their Implications in Adults With DLD

An important aspect of our findings is increased activation in bilateral caudate nucleus and putamen, collectively known as the striatum, in adults with DLD relative to adults with TD. The striatum, which is part of the basal ganglia, has been repeatedly implicated in DLD, but nearly all existing evidence comes from studies of children. Structural MRI work in children has shown reduced caudate nucleus volume, atypical maturation of the putamen, and altered microstructural properties in the basal ganglia, including reduced myelin content (Badcock et al., 2012; Cler et al., 2026; Krishnan et al., 2022; J. C. Lee et al., 2013; Soriano-Mas et al., 2009; Ullman et al., 2024). Functional studies in children likewise report atypical engagement of striatal circuits during language and procedural learning tasks (Badcock et al., 2012; Krishnan et al., 2021; Liégeois et al., 2014; Ullman et al., 2024). Together, this developmental literature has motivated theoretical accounts such as the Procedural Deficit Hypothesis (Ullman & Pierpont, 2005) as well as refinements to this theory (see Ullman et al., 2020, but also Cler et al., 2024, 2026), which attributes many language difficulties in DLD to atypical functioning of corticostriatal circuitry.

Our findings extend this work by demonstrating that striatal abnormalities persist into adulthood and are evident even during passive speech comprehension, a task that does not require procedural learning. This is notable because far less is known about the neural architecture of DLD in adults. Behavioral symptoms often attenuate or change form with age, and adults may develop compensatory strategies that mask underlying neurocognitive differences. The presence of increased caudate nucleus and putamen activation in our adult sample therefore provides evidence that striatal involvement in DLD is not limited to early developmental periods but remains a characteristic feature of the mature system.

The functional significance of this increased striatal activation in adults is not yet clear. One interpretation is that adults with DLD continue to rely disproportionately on procedural or learning-based corticostriatal circuits during speech processing, perhaps as a compensatory mechanism when cortical linguistic systems are less efficient. However, because our task did not require explicit learning, an alternative account is that elevated striatal activation reflects increased effort or reduced automaticity, mirroring the processing inefficiency described in younger populations (Badcock et al., 2012; Krishnan et al., 2021; Liégeois et al., 2003). A third possibility relates to microstructural findings in children. Reductions in myelin or atypical tissue properties in the striatum (particularly the caudate nucleus) have been observed in children with DLD (Bahar et al., 2023; Cler et al., 2026; Krishnan et al., 2022). If similar microstructural alterations persist into adulthood, increased functional recruitment may reflect compensatory neural activation required to achieve outcomes comparable to adults with TD. We consider the broader theoretical implications of these subcortical findings alongside the cortical and cerebellar results in a subsequent section (see the Toward a Mechanistic Framework of Brain and Behavior in DLD section).

### Neural Activation Patterns and Functional Implications in Adults With DLD

To better understand the neural mechanisms supporting language processing in adults with DLD, we compared activation patterns between groups and examined how these related to behavioral performance. Visual inspection of activation overlap maps revealed that although both groups recruited the expected frontotemporal language regions, adults with DLD showed a markedly reduced extent of activation in these areas, along with broader engagement of domain-general networks, again suggesting reduced efficiency within the specialized language network and greater reliance on general cognitive systems to support comprehension.

Individual differences further underscored this interpretation: Across both groups, stronger performance on the TILLS Sentence and Discourse Composite was associated with greater recruitment of left-hemisphere temporal regions. Within-group analyses revealed that this relationship was largely driven by variability within the DLD group, in which higher language scores were associated with a large left-hemisphere cluster encompassing canonical language regions, including the STG and MTG, IFG, and PT, as well as subcortical structures, including the thalamus, hippocampus, and putamen. No significant clusters emerged in the TD group, suggesting that language network recruitment may be more uniformly efficient in adults with TD and therefore less variable in relation to behavioral performance. The subcortical findings in the DLD group are interpreted cautiously, given the exploratory nature of these analyses, but may point to additional demands on corticosubcortical coordination in individuals whose cortical language systems are less efficiently organized.

Taken together, these findings support the view that stronger language outcomes in adults with DLD are associated with greater engagement of canonical frontotemporal language circuitry rather than diffuse or domain-general activation. However, we cannot rule out the possibility that the broader activation pattern observed in adults with DLD reflects lower baseline language abilities rather than active compensatory recruitment as reduced efficiency and diffuse activation may co-occur independently of any strategic reallocation of cognitive resources.

### Toward a Mechanistic Framework of Brain and Behavior in DLD

The cortical, cerebellar, and subcortical findings reported across the preceding sections, considered alongside the brain–behavior relationships described above, offer a preliminary basis for thinking about the neural organization of DLD in adulthood and its relationship to existing theoretical accounts. We now consider what these findings suggest collectively, with the aim of grounding them in a mechanistic framework that accounts for both brain and behavior in DLD, a gap we identified at the outset of this paper.

Theories of DLD such as the PDH (Ullman et al., 2020; Ullman & Pierpont, 2005) have been developed primarily in the context of childhood, and it remains unclear how well they extend to adulthood. The present findings offer a preliminary step toward filling this gap. While the subcortical differences we observed are broadly consistent with accounts implicating corticostriatal circuitry in DLD, including the PDH, our findings point to a more distributed pattern of atypical neural activation than any single framework predicts. Adults with DLD showed differences spanning cortical, subcortical, and cerebellar regions, alongside broader engagement of domain-general networks, suggesting that the neural consequences of DLD in adulthood are not reducible to disruption of a single circuit or system. Notably, our observation of hippocampal and thalamic involvement aligns with recent structural findings in children and adolescents with DLD (Cler et al., 2026), who reported smaller volumes in these regions in DLD as well as altered microstructure in the thalamus despite the PDH's prediction that medial-temporal lobe structures should be spared. Taken together, these findings challenge the view that subcortical differences in DLD are limited to the neostriatum.

We propose that these findings are better understood within a developmental cascade framework, in which early disruptions to language-relevant neural circuits, including but not limited to corticostriatal pathways, propagate over development into the broader pattern of atypical cortical recruitment and reduced neural efficiency we observe in adulthood. From this perspective, subcortical differences such as those observed in the caudate nucleus may represent a relatively stable endpoint of atypical development rather than necessarily a dynamic contributor to ongoing language performance, an interpretation supported by the fact that caudate nucleus differences at the group level were not associated with individual language scores in either group. In contrast, cortical and other subcortical regions including the putamen, thalamus, and hippocampus were associated with individual differences in language ability within the DLD group, suggesting that these regions continue to reflect meaningful variability in language outcomes even in adulthood. Together, this dissociation points to a system in which some neural differences may have become trait-like features of functional reorganization over development, while others retain sensitivity to individual variation in language ability.

This developmental cascade account is necessarily speculative, given the cross-sectional nature of the present data and the fact that the longitudinal evidence needed to test it directly does not yet exist. However, we believe that it represents a productive theoretical direction for bridging childhood and adult accounts of DLD and one that future longitudinal and multimodal imaging studies are well positioned to address. Importantly, these results also suggest that DLD represents a “difference” rather than “delay.” Even when language abilities become relatively strong in this population, underlying neural differences persist. That is, individuals with DLD may learn to use an atypical neural system to achieve complex operations, rather than follow a slowed but typical language and neural development trajectory.

### Limitations

Research including adults with DLD is very limited, and there are only a few studies that have investigated neural differences in adulthood; thus, in moving the field forward, there are a number of limitations related to participant characteristics and methodology that should be considered when interpreting the present findings and designing future studies.

First, identifying DLD in adults remains a major challenge. Unlike in childhood, standardized diagnostic tools for identifying DLD in adults are limited, and existing measures may not fully capture the subtle linguistic and cognitive difficulties that persist into adulthood. This is particularly relevant for bilingual adults, who may perform differently on standardized language assessments and demonstrate variable neural activation patterns depending on their language experience. Bilinguals were unevenly distributed across groups in our study (DLD: *n* = 3, 13%; TD: *n* = 8, 30%), and given known effects of bilingualism on language assessment performance and neural organization, this imbalance should be considered when interpreting group differences (Abutalebi, 2008; Bialystok & Feng, 2009). Even so, the lower language performance in the DLD group as a whole is unlikely to reflect differences in language exposure alone, given the higher percentage of bilinguals in the TD group. Relatedly, although the Fidler protocol provides a standardized and sensitive screening approach for adults (Fidler et al., 2011), it is possible that it identifies a broader profile of language-related weaknesses rather than DLD per se. In fact, several participants who met the Fidler criteria for DLD did not meet diagnostic thresholds on the TILLS, highlighting inconsistencies across available instruments. As noted by Choi-Tucci et al. (2024), increasing the cutoff score on the TILLS improves both sensitivity and specificity for adults, suggesting that diagnostic precision in adults may depend critically on how thresholds are defined. We note also that none of the subtests used for language characterization here across the Fidler protocol and TILLS have time constraints: All focus on whether the participant can complete a given task, not how much time (or effort, or alternative strategies) it may require. Findings from Hirn (2023) and Orrego et al. (2023) support the view that measures incorporating attention, processing speed, and cognitive effort may better differentiate adults with DLD from their typically developing peers. This aligns with the present findings, in which adults with DLD showed increased engagement of domain-general control networks, possibly reflecting greater processing effort. Future work should therefore explore composite diagnostic models that integrate both linguistic and non-linguistic measures of cognitive efficiency.

A related limitation concerns the exclusion of participants who reported a diagnosis of ADHD. Because ADHD is a neurodevelopmental condition that frequently co-occurs with DLD, excluding these individuals may limit the generalizability of our results. While this decision was made to isolate neural differences specific to DLD, future studies may benefit from including adults with ADHD in both groups and statistically controlling for ADHD symptoms as a covariate in the model. This approach could clarify whether the observed recruitment of domain-general systems reflects broader neurodevelopmental differences or language-specific compensatory mechanisms.

Additionally, our screening measures may have inadvertently selected for participants with domain-general processing differences. Tasks such as the modified token test require not only language processing but also sustained attention and working memory. Consequently, poorer performance on these screening measures could reflect attentional or memory deficits in addition to language impairment. If this occurred, the observed recruitment of domain-general neural systems during the passive listening task may partially reflect preexisting attentional differences between groups rather than language-specific compensatory mechanisms. However, this concern is somewhat mitigated by two things: (1) to be included in the DLD group, participants had to perform poorly on the spelling subtest of the Fidler, which likely does not reflect attention or working memory abilities; and (2) our finding that participants with stronger performance on the TILLS showed more typical patterns of neural activation in canonical language regions, such as the MTG, suggests that language ability does indeed contribute to the observed neural differences between groups.

Another consideration involves the task instructions for the MRI portion of the study. Participants were instructed to passively listen to the stimuli, but qualitative differences in how individuals interpreted these instructions may have influenced neural activation. Adults with DLD are often aware of their own language difficulties and thus may have adopted a more effortful or strategic listening approach (e.g., attempting to integrate isolated speech samples into a broader narrative), whereas adults with TD may have maintained a more passive stance. This could explain why individuals with DLD showed heightened activation in regions related to the multiple-demand and salience networks, potentially reflecting greater top-down control or monitoring, which would have important implications for how adults with DLD process language. Future studies could address this by including explicit task engagement measures (e.g., comprehension questions) or manipulating task demands to test this hypothesis directly.

A further limitation concerns the choice of control condition in the auditory language localizer. The acoustically degraded speech used here retains the suprasegmental properties of natural speech (e.g., rhythm, intonation, and amplitude envelope), which are absent in alternative control conditions such as reversed speech. Although Scott et al. (2017) argue that the precise nature of the control condition is unlikely to substantially affect the identification of high-level language regions, provided it lacks meaning and linguistic structure, it is possible that preserved suprasegmental cues in the degraded condition differentially engaged lower-level speech processing regions across groups. In particular, the MTG activation observed in the DLD group may partly reflect greater effort to extract meaning from the degraded stimuli rather than differences in high-level language processing per se. Future studies may benefit from including other control conditions such as reversed speech to disentangle contributions of suprasegmental processing from higher-level linguistic processing and to more precisely characterize group differences in speech perception.

Our choice of statistical thresholds may have also influenced the apparent group differences in spatial extent of activation. It is possible that adults with DLD activated similar language regions as adults with TD but with weaker signal strength that fell below the cluster-defining threshold (*z* > 3.1). More fine-grained analyses, such as ROI comparisons or connectivity-based approaches could reveal subtler group differences in activation magnitude or network dynamics that were not captured in the present whole-brain contrasts.

It is also worth noting that the language localizer approach has been critiqued for its reliance on localizationist assumptions that may not capture the full extent of distributed language processing and for the risk of circular inference when localizer-defined regions are used to constrain subsequent analyses of the same data (Grodzinsky, 2010; Murphy & Woolnough, 2024). Importantly, however, our use of this task differed from its typical application. Rather than using it to functionally define ROIs for subsequent analyses, we treated it as a standard language task, where we analyzed activation at the individual level and compared patterns across groups. We view the localizer as a useful starting point for characterizing the functional language network in a population with extremely limited existing neuroimaging evidence.

Finally, it is important to acknowledge that the gender distribution in our sample differs from what is typically reported in childhood DLD, where males are diagnosed nearly twice as often as females (Chilosi et al., 2023), though some population-based studies suggest the true prevalence is closer to 1:1 (Norbury et al., 2016). In the present study, women were overrepresented in both groups (DLD: 18 women, four men, 1 nonbinary; TD: 22 women, 5 men), a pattern consistent with our broader experience that women volunteer for research more readily than men. Across all of our experiments over a 3-year period, 259 participants completed at least one study and reported a gender or sex; of these, 182 (70.3%) were women, 64 (24.7%) were men, and 13 (5%) identified as nonbinary or genderqueer. Our lab does not restrict participation by gender or sex, nor is gender recorded until participation is complete. Importantly, the comparable gender distribution across groups (DLD: 78% women; TD: 81% women) suggests that gender is unlikely to account for the observed between-groups differences. Whether the neural profiles reported here are partly shaped by sex- or gender-related factors remains an open question for future research.

Despite these caveats, the current study benefits from a relatively large sample and a well-validated language localizer, lending confidence to the observed patterns of network recruitment and their interpretation.

### Conclusion

Collectively, the present findings advance our understanding of the neural substrates supporting language in adults with DLD. This study provides new evidence that adults with DLD, despite broadly intact auditory perception, engage less efficient and more diffuse neural pathways to support language comprehension. Rather than relying primarily on the specialized frontotemporal language network, they appear to recruit additional domain-general systems, potentially reflecting compensatory but cognitively costly strategies. These findings highlight that persistent differences in neural organization can underlie subtle language weaknesses, even when overt performance appears typical. This discrepancy between behavior and neural activation also highlights the real-world communication challenges that adults with DLD face. Increased reliance on executive and subcortical systems may serve as a hidden indicator of effort, revealing differences in processing efficiency, attentional mechanisms, and/or susceptibility to fatigue that may only surface in more demanding or ecologically complex contexts. Overall, this research points to the importance of considering large-scale network interactions in models of DLD. Future research should build on these findings by including longitudinal and multimodal imaging to characterize how compensatory mechanisms emerge and evolve from childhood into adulthood in individuals with DLD, as well as examining the precise nature in which language abilities and brain function differs between adults with and without DLD.

## ACKNOWLEDGMENTS

The authors thank Iris Mendoza Luna, Anja Bullen, and Allison Johnson for their assistance with data collection and the team members of the University of Washington Center for Human Neuroscience for their assistance with the setup of the MRI sequences and data acquisition. They also extend their sincere gratitude to all the participants who took part in this study and to the reviewers for their thoughtful and constructive comments. Finally, they thank Paula Orrego and Juliana Hirn for their guidance and insight into their experiences living with DLD.

## FUNDING INFORMATION

Linnea Beasley, University of Washington Center for Human Neuroscience, Student Technology Fee. Noelle T. Abbott, National Institute on Deafness and Other Communication Disorders (https://dx.doi.org/10.13039/100000055), Award ID: T32DC005361-24. Noelle T. Abbott, Virginia Merrill Bloedel Trainee Award.

## AUTHOR CONTRIBUTIONS

**Noelle T. Abbott**: Conceptualization: Supporting; Data curation: Equal; Formal analysis: Lead; Funding acquisition: Equal; Investigation: Equal; Methodology: Equal; Project administration: Equal; Validation: Lead; Visualization: Lead; Writing – original draft: Lead; Writing – review & editing: Equal. **Linnea Beasley**: Data curation: Equal; Funding acquisition: Supporting. **Gabriel J. Cler**: Conceptualization: Lead; Funding acquisition: Supporting; Methodology: Lead; Resources: Lead; Supervision: Lead; Writing – review & editing: Lead.

## DATA AND CODE AVAILABILITY STATEMENTS

The MRI data that support the findings of this study are openly available in Neurovault at https://identifiers.org/neurovault.collection:23664. The de-identified participant scores and associated R code are available at https://osf.io/rzb96/overview?view_only=470093de422a455d9b1efd8ca540cef1.

## Supplementary Material



## TECHNICAL TERMS

Developmental language disorder (DLD): A neurodevelopmental condition characterized by persistent difficulties in language comprehension and production that cannot be explained by other factors.
Functional language localizer: An fMRI task designed to functionally identify language-responsive brain regions in individual participants by contrasting linguistic and non-linguistic conditions.

## References

[bib1] Abbott, N., & Love, T. (2023). Bridging the divide: Brain and behavior in developmental language disorder. Brain Sciences, 13(11), 1606. 10.3390/brainsci13111606, 38002565 PMC10670267

[bib2] Abutalebi, J. (2008). Neural aspects of second language representation and language control. Acta Psychologica, 128(3), 466–478. 10.1016/j.actpsy.2008.03.014, 18479667

[bib3] Andersson, J. L. R., Jenkinson, M., & Smith, S. (2019). High resolution nonlinear registration with simultaneous modelling of intensities. bioRxiv. 10.1101/646802

[bib4] Andersson, J. L. R., Skare, S., & Ashburner, J. (2003). How to correct susceptibility distortions in spin-echo echo-planar images: Application to diffusion tensor imaging. NeuroImage, 20(2), 870–888. 10.1016/S1053-8119(03)00336-7, 14568458

[bib5] Badcock, N. A., Bishop, D. V. M., Hardiman, M. J., Barry, J. G., & Watkins, K. E. (2012). Co-localisation of abnormal brain structure and function in specific language impairment. Brain and Language, 120(3), 310–320. 10.1016/j.bandl.2011.10.006, 22137677 PMC3315677

[bib6] Bahar, N., Cler, G. J., Krishnan, S., Asaridou, S. S., Smith, H. J., Willis, H. E., Healy, M. P., & Watkins, K. E. (2023). Differences in cortical surface area in developmental language disorder. Neurobiology of Language, 5(2), 288–314. 10.1162/nol_a_00127, 38832358 PMC11093399

[bib7] Bautista, A., & Wilson, S. M. (2016). Neural responses to grammatically and lexically degraded speech. Language, Cognition and Neuroscience, 31(4), 567–574. 10.1080/23273798.2015.1123281, 27525290 PMC4981484

[bib8] Becker, T. C., & McGregor, K. K. (2016). Learning by listening to lectures is a challenge for college students with developmental language impairment. Journal of Communication Disorders, 64, 32–44. 10.1016/j.jcomdis.2016.09.001, 27721078 PMC6540751

[bib9] Bethlehem, R. A. I., Seidlitz, J., White, S. R., Vogel, J. W., Anderson, K. M., Adamson, C., Adler, S., Alexopoulos, G. S., Anagnostou, E., Areces-Gonzalez, A., Astle, D. E., Auyeung, B., Ayub, M., Bae, J., Ball, G., Baron-Cohen, S., Beare, R., Bedford, S. A., Benegal, V., … Alexander-Bloch, A. F. (2022). Brain charts for the human lifespan. Nature, 604(7906), 525–533. 10.1038/s41586-022-04554-y, 35388223 PMC9021021

[bib10] Bialystok, E., & Feng, X. (2009). Language proficiency and executive control in proactive interference: Evidence from monolingual and bilingual children and adults. Brain and Language, 109(2–3), 93–100. 10.1016/j.bandl.2008.09.001, 18834625 PMC2699211

[bib11] Billot, A., Jhingan, N., Varkanitsa, M., Blank, I., Ryskin, R., Kiran, S., & Fedorenko, E. (2024). The language network ages well: Preserved selectivity, lateralization, and within-network functional synchronization in older brains. bioRxiv. 10.1101/2024.10.23.619954, 42637780 PMC13503826

[bib12] Bishop, D. V. M. (2017). Why is it so hard to reach agreement on terminology? The case of developmental language disorder (DLD). International Journal of Language & Communication Disorders, 52(6), 671–680. 10.1111/1460-6984.12335, 28714100 PMC5697617

[bib13] Bishop, D. V. M., Snowling, M. J., Thompson, P. A., Greenhalgh, T., & the CATALISE-2 consortium. (2017). Phase 2 of CATALISE: A multinational and multidisciplinary Delphi consensus study of problems with language development: Terminology. Journal of Child Psychology and Psychiatry, 58(10), 1068–1080. 10.1111/jcpp.12721, 28369935 PMC5638113

[bib14] Botting, N. (2020). Language, literacy and cognitive skills of young adults with developmental language disorder (DLD). International Journal of Language & Communication Disorders, 55(2), 255–265. 10.1111/1460-6984.12518, 31994284

[bib15] Briggs, R. G., Lin, Y.-H., Dadario, N. B., Kim, S. J., Young, I. M., Bai, M. Y., Dhanaraj, V., Fonseka, R. D., Hormovas, J., Tanglay, O., Chakraborty, A. R., Milligan, T. M., Abraham, C. J., Anderson, C. D., Palejwala, A. H., Conner, A. K., O’Donoghue, D. L., & Sughrue, M. E. (2021). Anatomy and white matter connections of the middle frontal gyrus. World Neurosurgery, 150, e520–e529. 10.1016/j.wneu.2021.03.045, 33744423

[bib16] Bryłka, M., Wojciechowski, J., Wolak, T., & Cygan, H. B. (2025). Compensatory mechanisms in visual sequence learning: An fMRI study of children with developmental language disorder. bioRxiv. 10.1101/2025.06.16.659875 41547519

[bib17] Buckner, R. L., Andrews-Hanna, J. R., & Schacter, D. L. (2008). The brain’s default network: Anatomy, function, and relevance to disease. Annals of the New York Academy of Sciences, 1124(1), 1–38. 10.1196/annals.1440.011, 18400922

[bib18] Carrow-Woolfolk, E. (2017). Comprehensive assessment of spoken language (2nd ed., Test Easel 3: Supralinguistic and pragmatic tests). Western Psychological Services.

[bib19] Chilosi, A. M., Brovedani, P., Cipriani, P., & Casalini, C. (2023). Sex differences in early language delay and in developmental language disorder. Journal of Neuroscience Research, 101(5), 654–667. 10.1002/jnr.24976, 34822733

[bib20] Choi-Tucci, A., White, M., & Plante, E. (2024). Determining the diagnostic accuracy of the Test of Integrated Language and Literacy Skills for college students. American Journal of Speech-Language Pathology, 33(2), 1051–1058. 10.1044/2023_AJSLP-23-00331, 38118454

[bib21] Clegg, J., Hollis, C., Mawhood, L., & Rutter, M. (2005). Developmental language disorders—A follow-up in later adult life. Cognitive, language and psychosocial outcomes. Journal of Child Psychology and Psychiatry, 46(2), 128–149. 10.1111/j.1469-7610.2004.00342.x, 15679523

[bib22] Cler, G. J., Asaridou, S. S., Bahar, N., Krishnan, S., Smith, H. J., Willis, H. E., Healy, M. P., & Watkins, K. E. (2026). Subcortical correlates of developmental language disorder: More than the neostriatum. Brain Communications, 8(1), fcaf493. 10.1093/braincomms/fcaf493, 41550306 PMC12809564

[bib23] Cler, G. J., Bartolo, S., Kim, J., Nolan, A., & Banel, S. (2024). Implicit and explicit sequence learning in adults with developmental language disorder. Journal of Speech, Language, and Hearing Research, 67(8), 2638–2652. 10.1044/2024_JSLHR-23-00551, 39024517

[bib24] Conti-Ramsden, G., Durkin, K., Toseeb, U., Botting, N., & Pickles, A. (2017). Education and employment outcomes of young adults with a history of developmental language disorder. International Journal of Language & Communication Disorders, 53(2), 237–255. 10.1111/1460-6984.12338, 29139196 PMC5873379

[bib25] De Fossé, L., Hodge, S. M., Makris, N., Kennedy, D. N., Caviness, V. S., McGrath, L., Steele, S., Ziegler, D. A., Herbert, M. R., Frazier, J. A., Tager-Flusberg, H., & Harris, G. J. (2004). Language-association cortex asymmetry in autism and specific language impairment. Annals of Neurology, 56(6), 757–766. 10.1002/ana.20275, 15478219

[bib26] de Guibert, C., Maumet, C., Jannin, P., Ferré, J.-C., Tréguier, C., Barillot, C., Le Rumeur, E., Allaire, C., & Biraben, A. (2011). Abnormal functional lateralization and activity of language brain areas in typical specific language impairment (developmental dysphasia). Brain, 134(10), 3044–3058. 10.1093/brain/awr141, 21719430 PMC5331119

[bib27] Del Tufo, S. N., & Earle, F. S. (2020). Skill profiles of college students with a history of developmental language disorder and developmental dyslexia. Journal of Learning Disabilities, 53(3), 228–240. 10.1177/0022219420904348, 32028829 PMC7336274

[bib28] Doucet, G. E., Kruse, J. A., Mertens, A., Goldsmith, C., Eden, N. M., Oleson, J., & McGregor, K. K. (2025). Subcortical brain iron and its link to verbal memory in children with developmental language disorder. Brain and Language, 261, 105531. 10.1016/j.bandl.2024.105531, 39756358 PMC11769726

[bib29] Doucet, G. E., Kruse, J. A., Eden, N. M., Goffman, L., & McGregor, K. K. (2025). Initial evidence of altered functional network connectivity in children with developmental language disorder. Brain and Language, 270, 105637. 10.1016/j.bandl.2025.105637, 40886422 PMC12690564

[bib30] Dubois, P., St-Pierre, M.-C., Desmarais, C., & Guay, F. (2020). Young adults with developmental language disorder: A systematic review of education, employment, and independent living outcomes. Journal of Speech, Language, and Hearing Research, 63(11), 3786–3800. 10.1044/2020_JSLHR-20-00127, 33022192

[bib31] Erb, J., Henry, M. J., Eisner, F., & Obleser, J. (2013). The brain dynamics of rapid perceptual adaptation to adverse listening conditions. Journal of Neuroscience, 33(26), 10688–10697. 10.1523/JNEUROSCI.4596-12.2013, 23804092 PMC6618499

[bib32] Estes, K. G., Evans, J. L., & Else-Quest, N. M. (2007). Differences in the nonword repetition performance of children with and without specific language impairment: A meta-analysis. Journal of Speech, Language, and Hearing Research, 50(1), 177–195. 10.1044/1092-4388(2007/015), 17344558

[bib33] Fairbanks, G. (1960). Voice and articulation drillbook (2nd ed.). Harper. https://cir.nii.ac.jp/crid/1970867909805291586

[bib34] Fassbender, C., Simoes-Franklin, C., Murphy, K., Hester, R., Meaney, J., Robertson, I. H., & Garavan, H. (2006). The role of a right fronto-parietal network in cognitive control: Common activations for “cues-to-attend” and response inhibition. Journal of Psychophysiology, 20(4), 286–296. 10.1027/0269-8803.20.4.286

[bib35] Fedorenko, E., Behr, M. K., & Kanwisher, N. (2011). Functional specificity for high-level linguistic processing in the human brain. Proceedings of the National Academy of Sciences, 108(39), 16428–16433. 10.1073/pnas.1112937108, 21885736 PMC3182706

[bib36] Fedorenko, E., Duncan, J., & Kanwisher, N. (2013). Broad domain generality in focal regions of frontal and parietal cortex. Proceedings of the National Academy of Sciences, 110(41), 16616–16621. 10.1073/pnas.1315235110, 24062451 PMC3799302

[bib37] Fedorenko, E., Hsieh, P.-J., Nieto-Castañón, A., Whitfield-Gabrieli, S., & Kanwisher, N. (2010). New method for fMRI investigations of language: Defining ROIs functionally in individual subjects. Journal of Neurophysiology, 104(2), 1177–1194. 10.1152/jn.00032.2010, 20410363 PMC2934923

[bib38] Fedorenko, E., Ivanova, A. A., & Regev, T. I. (2024). The language network as a natural kind within the broader landscape of the human brain. Nature Reviews Neuroscience, 25(5), 289–312. 10.1038/s41583-024-00802-4, 38609551 PMC13222024

[bib39] Fidler, L. J., Plante, E., & Vance, R. (2011). Identification of adults with developmental language impairments. American Journal of Speech-Language Pathology, 20(1), 2–13. 10.1044/1058-0360(2010/09-0096), 20739630

[bib40] Gathercole, S. E., & Baddeley, A. D. (1990). Phonological memory deficits in language disordered children: Is there a causal connection? Journal of Memory and Language, 29(3), 336–360. 10.1016/0749-596X(90)90004-J

[bib41] Gauger, L. M., Lombardino, L. J., & Leonard, C. M. (1997). Brain morphology in children with specific language impairment. Journal of Speech Language and Hearing Research, 40(6), 1272–1284. 10.1044/jslhr.4006.1272, 9430748

[bib42] Goulden, N., Khusnulina, A., Davis, N. J., Bracewell, R. M., Bokde, A. L., McNulty, J. P., & Mullins, P. G. (2014). The salience network is responsible for switching between the default mode network and the central executive network: Replication from DCM. NeuroImage, 99, 180–190. 10.1016/j.neuroimage.2014.05.052, 24862074

[bib43] Greve, D. N., & Fischl, B. (2009). Accurate and robust brain image alignment using boundary-based registration. NeuroImage, 48(1), 63–72. 10.1016/j.neuroimage.2009.06.060, 19573611 PMC2733527

[bib44] Grodzinsky, Y. (2010). The picture of the linguistic brain: How sharp can it be? Reply to Fedorenko & Kanwisher. Language and Linguistics Compass, 4(8), 605–622. 10.1111/j.1749-818X.2010.00222.x, 20976129 PMC2957117

[bib45] Grodzinsky, Y., & Amunts, K. (Eds.). (2006). Broca’s region. Oxford University Press. 10.1093/acprof:oso/9780195177640.001.0001

[bib46] Grunow, H., Spaulding, T. J., Gómez, R. L., & Plante, E. (2006). The effects of variation on learning word order rules by adults with and without language-based learning disabilities. Journal of Communication Disorders, 39(2), 158–170. 10.1016/j.jcomdis.2005.11.004, 16376369

[bib47] Hall, J., McGregor, K., & Oleson, J. (2017). Weaknesses in lexical-semantic knowledge among college students with specific learning disabilities: Evidence from a semantic fluency task. Journal of Speech, Language, and Hearing Research, 60(3), 640–653. 10.1044/2016_JSLHR-L-15-0440, 28267833 PMC5544191

[bib48] Hernández, D., Kärkkäinen, S., Tulonen, T., Helenius, P., Salmelin, R., & Parviainen, T. (2022). Attentional modulation of interhemispheric (a)symmetry in children with developmental language disorder. Scientific Reports, 12(1), 17904. 10.1038/s41598-022-22820-x, 36284164 PMC9596496

[bib49] Hickok, G., & Poeppel, D. (2007). The cortical organization of speech processing. Nature Reviews Neuroscience, 8(5), 393–402. 10.1038/nrn2113, 17431404

[bib50] Hickok, G., & Poeppel, D. (2016). Neural basis of speech perception. In G. Hickok & S. L. Small (Eds.), Neurobiology of language (pp. 299–310). Elsevier. 10.1016/B978-0-12-407794-2.00025-0

[bib51] Hirn, J. (2023). From my perspective/opinion: What it’s like being an SLP with DLD. Leader Live. https://leader.pubs.asha.org/do/10.1044/leader.FMP.28092023.slp-dld-advocacy.8/full/

[bib52] Hugdahl, K., Gundersen, H., Brekke, C., Thomsen, T., Rimol, L. M., Ersland, L., & Niemi, J. (2004). fMRI brain activation in a Finnish family with specific language impairment compared with a normal control group. Journal of Speech, Language, and Hearing Research, 47(1), 162–172. 10.1044/1092-4388(2004/014), 15072536

[bib53] Isaki, E., & Plante, E. (1997). Short-term and working memory differences in language/learning disabled and normal adults. Journal of Communication Disorders, 30(6), 427–436. 10.1016/S0021-9924(96)00111-6, 9397387

[bib54] Jenkinson, M., Bannister, P., Brady, M., & Smith, S. (2002). Improved optimization for the robust and accurate linear registration and motion correction of brain images. NeuroImage, 17(2), 825–841. 10.1006/nimg.2002.1132, 12377157

[bib55] Jernigan, T. L., Hesselink, J. R., Sowell, E., & Tallal, P. A. (1991). Cerebral structure on magnetic resonance imaging in language- and learning-impaired children. Archives of Neurology, 48(5), 539–545. 10.1001/archneur.1991.00530170103028, 2021369

[bib56] Jouravlev, O., Kell, A. J. E., Mineroff, Z., Haskins, A. J., Ayyash, D., Kanwisher, N., & Fedorenko, E. (2020). Reduced language lateralization in autism and the broader autism phenotype as assessed with robust individual-subjects analyses. Autism Research, 13(10), 1746–1761. 10.1002/aur.2393, 32935455

[bib57] Kast, M., Bezzola, L., Jäncke, L., & Meyer, M. (2011). Multi- and unisensory decoding of words and nonwords result in differential brain responses in dyslexic and nondyslexic adults. Brain and Language, 119(3), 136–148. 10.1016/j.bandl.2011.04.002, 21641022

[bib58] Krishnan, S., Asaridou, S. S., Cler, G. J., Smith, H. J., Willis, H. E., Healy, M. P., Thompson, P. A., Bishop, D. V. M., & Watkins, K. E. (2021). Functional organisation for verb generation in children with developmental language disorder. NeuroImage, 226, 117599. 10.1016/j.neuroimage.2020.117599, 33285329 PMC7836232

[bib59] Krishnan, S., Cler, G. J., Smith, H. J., Willis, H. E., Asaridou, S. S., Healy, M. P., Papp, D., & Watkins, K. E. (2022). Quantitative MRI reveals differences in striatal myelin in children with DLD. eLife, 11, e74242. 10.7554/eLife.74242, 36164824 PMC9514847

[bib60] Kurth, F., Luders, E., Pigdon, L., Conti-Ramsden, G., Reilly, S., & Morgan, A. T. (2018). Altered gray matter volumes in language-associated regions in children with developmental language disorder and speech sound disorder. Developmental Psychobiology, 60(7), 814–824. 10.1002/dev.21762, 30101474

[bib61] Lee, J. C., Dick, A. S., & Tomblin, J. B. (2020). Altered brain structures in the dorsal and ventral language pathways in individuals with and without developmental language disorder (DLD). Brain Imaging and Behavior, 14(6), 2569–2586. 10.1007/s11682-019-00209-1, 31933046 PMC7354888

[bib62] Lee, J. C., Nopoulos, P. C., & Bruce Tomblin, J. (2013). Abnormal subcortical components of the corticostriatal system in young adults with DLI: A combined structural MRI and DTI study. Neuropsychologia, 51(11), 2154–2161. 10.1016/j.neuropsychologia.2013.07.011, 23896446 PMC3816105

[bib63] Lee, J. J. (2022). Efficient localization of the cortical language network and its functional neuroanatomy in dyslexia [Doctoral dissertation, Boston University]. https://www.proquest.com/docview/2628740068/abstract/90E40440D36546B3PQ/1

[bib64] Lee, J. J., Scott, T. L., & Perrachione, T. K. (2024). Efficient functional localization of language regions in the brain. NeuroImage, 285, 120489. 10.1016/j.neuroimage.2023.120489, 38065277 PMC10999251

[bib65] Leonard, L. B. (2014). Children with specific language impairment and their contribution to the study of language development. Journal of Child Language, 41(S1), 38–47. 10.1017/s0305000914000130, 25023495 PMC4429873

[bib66] Leung, H.-C., Gore, J. C., & Goldman-Rakic, P. S. (2002). Sustained mnemonic response in the human middle frontal gyrus during on-line storage of spatial memoranda. Journal of Cognitive Neuroscience, 14(4), 659–671. 10.1162/08989290260045882, 12126506

[bib67] Li, H., Booth, J. R., Feng, X., Wei, N., Zhang, M., Zhang, J., Zhong, H., Lu, C., Liu, L., Ding, G., & Meng, X. (2020). Functional parcellation of the right cerebellar lobule VI in children with normal or impaired reading. Neuropsychologia, 148, 107630. 10.1016/j.neuropsychologia.2020.107630, 32976851 PMC7704787

[bib68] Liégeois, F., Baldeweg, T., Connelly, A., Gadian, D. G., Mishkin, M., & Vargha-Khadem, F. (2003). Language fMRI abnormalities associated with FOXP2 gene mutation. Nature Neuroscience, 6(11), 1230–1237. 10.1038/nn1138, 14555953

[bib69] Liégeois, F., Mayes, A., & Morgan, A. (2014). Neural correlates of developmental speech and language disorders: Evidence from neuroimaging. Current Developmental Disorders Reports, 1(3), 215–227. 10.1007/s40474-014-0019-1, 25057455 PMC4104164

[bib70] Liu, J. R., Zhao, L., Hullett, P. W., & Chang, E. F. (2025). Speech sequencing in the human precentral gyrus. Nature Human Behaviour, 9(11), 2327–2344. 10.1038/s41562-025-02250-1, 40670697 PMC13525832

[bib71] Malik-Moraleda, S., Ayyash, D., Gallée, J., Affourtit, J., Hoffmann, M., Mineroff, Z., Jouravlev, O., & Fedorenko, E. (2022). An investigation across 45 languages and 12 language families reveals a universal language network. Nature Neuroscience, 25(8), 1014–1019. 10.1038/s41593-022-01114-5, 35856094 PMC10414179

[bib72] Malins, J. G., Gumkowski, N., Buis, B., Molfese, P., Rueckl, J. G., Frost, S. J., Pugh, K. R., Morris, R., & Mencl, W. E. (2016). Dough, tough, cough, rough: A “fast” fMRI localizer of component processes in reading. Neuropsychologia, 91, 394–406. 10.1016/j.neuropsychologia.2016.08.027, 27592331 PMC5089170

[bib73] Mansouri, F. A., Koechlin, E., Rosa, M. G. P., & Buckley, M. J. (2017). Managing competing goals—A key role for the frontopolar cortex. Nature Reviews Neuroscience, 18(11), 645–657. 10.1038/nrn.2017.111, 28951610

[bib74] McGregor, K. K. (2020). How we fail children with developmental language disorder. Language, Speech, and Hearing Services in Schools, 51(4), 981–992. 10.1044/2020_LSHSS-20-00003, 32755505 PMC7842848

[bib75] McGregor, K., Arbisi-Kelm, T., & Eden, N. (2017). The encoding of word forms into memory may be challenging for college students with developmental language impairment. International Journal of Speech-Language Pathology, 19(1), 43–57. 10.3109/17549507.2016.1159337, 27068639 PMC5471608

[bib76] McGregor, K. K., Eden, N., Arbisi-Kelm, T., & Oleson, J. (2020). The fast-mapping abilities of adults with developmental language disorder. Journal of Speech, Language, and Hearing Research, 63(9), 3117–3129. 10.1044/2020_JSLHR-19-00418, 32787708 PMC7890218

[bib77] McGregor, K. K., Gordon, K., Eden, N., Arbisi-Kelm, T., & Oleson, J. (2017). Encoding deficits impede word learning and memory in adults with developmental language disorders. Journal of Speech, Language, and Hearing Research, 60(10), 2891–2905. 10.1044/2017_JSLHR-L-17-0031, 28980007 PMC5945064

[bib78] Miller, C. A., & Poll, G. H. (2009). Response time in adults with a history of language difficulties. Journal of Communication Disorders, 42(5), 365–379. 10.1016/j.jcomdis.2009.04.001, 19428024

[bib79] Mineroff, Z., Blank, I. A., Mahowald, K., & Fedorenko, E. (2018). A robust dissociation among the language, multiple demand, and default mode networks: Evidence from inter-region correlations in effect size. Neuropsychologia, 119, 501–511. 10.1016/j.neuropsychologia.2018.09.011, 30243926 PMC6191329

[bib80] Murphy, E., & Woolnough, O. (2024). The language network is topographically diverse and driven by rapid syntactic inferences. Nature Reviews Neuroscience, 25(10), 705. 10.1038/s41583-024-00852-8, 39123048

[bib81] Nakatani, H., Nakamura, Y., & Okanoya, K. (2023). Respective involvement of the right cerebellar crus I and II in syntactic and semantic processing for comprehension of language. Cerebellum, 22(4), 739–755. 10.1007/s12311-022-01451-y, 35927417

[bib82] Nelson, N., Plante, E., Helm-Estabrooks, N., & Hotz, G. (2016). Test of Integrated Language and Literacy Skills (TILLS) technical manual. Brookes Publishing.

[bib83] Norbury, C. F., Gooch, D., Wray, C., Baird, G., Charman, T., Simonoff, E., Vamvakas, G., & Pickles, A. (2016). The impact of nonverbal ability on prevalence and clinical presentation of language disorder: Evidence from a population study. Journal of Child Psychology and Psychiatry, 57(11), 1247–1257. 10.1111/jcpp.12573, 27184709 PMC5082564

[bib84] Norman-Haignere, S., Kanwisher, N. G., & McDermott, J. H. (2015). Distinct cortical pathways for music and speech revealed by hypothesis-free voxel decomposition. Neuron, 88(6), 1281–1296. 10.1016/j.neuron.2015.11.035, 26687225 PMC4740977

[bib85] Oberhuber, M., Hope, T. M. H., Seghier, M. L., Parker Jones, O., Prejawa, S., Green, D. W., & Price, C. J. (2016). Four functionally distinct regions in the left supramarginal gyrus support word processing. Cerebral Cortex, 26(11), 4212–4226. 10.1093/cercor/bhw251, 27600852 PMC5066832

[bib86] Obleser, J., & Kotz, S. A. (2010). Expectancy constraints in degraded speech modulate the language comprehension network. Cerebral Cortex, 20(3), 633–640. 10.1093/cercor/bhp128, 19561061

[bib87] Orrego, P. M., McGregor, K. K., & Reyes, S. M. (2023). A first-person account of developmental language disorder. American Journal of Speech-Language Pathology, 32(4), 1383–1396. 10.1044/2023_AJSLP-22-00247, 37195674 PMC10473366

[bib88] Pigdon, L., Willmott, C., Reilly, S., Conti-Ramsden, G., Liegeois, F., Connelly, A., & Morgan, A. T. (2020). The neural basis of nonword repetition in children with developmental speech or language disorder: An fMRI study. Neuropsychologia, 138, 107312 10.1016/j.neuropsychologia.2019.107312, 31917203

[bib89] Plante, E. (1991). MRI findings in the parents and siblings of specifically language-impaired boys. Brain and Language, 41(1), 67–80. 10.1016/0093-934X(91)90111-D, 1884192

[bib90] Plante, E., Gomez, R., & Gerken, L. A. (2002). Sensitivity to word order cues by normal and language/learning disabled adults. Journal of Communication Disorders, 35(5), 453–462. 10.1016/S0021-9924(02)00094-1, 12194564

[bib91] Plante, E., Patterson, D., Sandoval, M., Vance, C. J., & Asbjørnsen, A. E. (2017). An fMRI study of implicit language learning in developmental language impairment. NeuroImage: Clinical, 14, 277–285. 10.1016/j.nicl.2017.01.027, 28203531 PMC5295640

[bib92] Plante, E., Ramage, A. E., & Magloire, J. (2006). Processing narratives for verbatim and gist information by adults with language learning disabilities: A functional neuroimaging study. Learning Disabilities Research & Practice, 21(1), 61–76. 10.1111/j.1540-5826.2006.00207.x

[bib93] Poll, G. H., Betz, S. K., & Miller, C. A. (2010). Identification of clinical markers of specific language impairment in adults. Journal of Speech, Language, and Hearing Research, 53(2), 414–429. 10.1044/1092-4388(2009/08-0016), 20360465

[bib94] Poll, G. H., Brown, B., & Miller, C. A. (2025). Exploring the effect of sentence structure frequency on the accuracy of a screener for adults at risk of developmental language disorder. Journal of Speech, Language, and Hearing Research, 68(7), 3348–3356. 10.1044/2025_JSLHR-24-00628, 40489643

[bib95] Poll, G. H., & Martin, A. (2022). Moment-to-moment processing of complex sentences by adults with and without developmental language disorder. Journal of Communication Disorders, 99, 106258. 10.1016/j.jcomdis.2022.106258, 36029614 PMC9536528

[bib96] Poll, G. H., & Miller, C. A. (2021). Speech production factors and verbal working memory in children and adults with developmental language disorder. Applied Psycholinguistics, 42(3), 673–702. 10.1017/S0142716421000011, 34024959 PMC8135931

[bib97] Poll, G. H., Miller, C. A., & van Hell, J. G. (2015). Evidence of compensatory processing in adults with developmental language impairment: Testing the predictions of the procedural deficit hypothesis. Journal of Communication Disorders, 53, 84–102. 10.1016/j.jcomdis.2015.01.004, 25628150 PMC4346385

[bib98] Poll, G. H., Miller, C. A., & van Hell, J. G. (2016). Sentence repetition accuracy in adults with developmental language impairment: Interactions of participant capacities and sentence structures. Journal of Speech, Language, and Hearing Research, 59(2), 302–316. 10.1044/2015_JSLHR-L-15-0020, 27272196 PMC4972009

[bib99] Poll, G. H., Watkins, H. S., & Miller, C. A. (2014). Lexical decay during online sentence processing in adults with specific language impairment. Journal of Speech, Language, and Hearing Research, 57(6), 2253–2260. 10.1044/2014_JSLHR-L-13-0265, 25104299

[bib100] Price, C. J. (2010). The anatomy of language: A review of 100 fMRI studies published in 2009. Annals of the New York Academy of Sciences, 1191(1), 62–88. 10.1111/j.1749-6632.2010.05444.x, 20392276

[bib101] Raichle, M. E., MacLeod, A. M., Snyder, A. Z., Powers, W. J., Gusnard, D. A., & Shulman, G. L. (2001). A default mode of brain function. Proceedings of the National Academy of Sciences, 98(2), 676–682. 10.1073/pnas.98.2.676, 11209064 PMC14647

[bib102] Records, N. L., Tomblin, J. B., & Buckwalter, P. R. (1995). Auditory verbal learning and memory in young adults with specific language impairment. Clinical Neuropsychologist, 9(2), 187–193. 10.1080/13854049508401601

[bib103] Richardson, J., Harris, L., Plante, E., & Gerken, L. (2006). Subcategory learning in normal and language learning-disabled adults: How much information do they need? Journal of Speech, Language, and Hearing Research, 49(6), 1257–1266. 10.1044/1092-4388(2006/090), 17197494

[bib104] Richlan, F., Kronbichler, M., & Wimmer, H. (2011). Meta-analyzing brain dysfunctions in dyslexic children and adults. NeuroImage, 56(3), 1735–1742. 10.1016/j.neuroimage.2011.02.040, 21338695

[bib105] Rissman, J., Eliassen, J. C., & Blumstein, S. E. (2003). An event-related fMRI investigation of implicit semantic priming. Journal of Cognitive Neuroscience, 15(8), 1160–1175. 10.1162/089892903322598120, 14709234

[bib106] Roberts, T. P. L., Heiken, K., Zarnow, D., Dell, J., Nagae, L., Blaskey, L., Solot, C., Levy, S. E., Berman, J. I., & Edgar, J. C. (2014). Left hemisphere diffusivity of the arcuate fasciculus: Influences of autism spectrum disorder and language impairment. American Journal of Neuroradiology, 35(3), 587–592. 10.3174/ajnr.A3754, 24335547 PMC3970288

[bib107] Rosselli, M., Ardila, A., Matute, E., & Vélez-Uribe, I. (2014). Language development across the life span: A neuropsychological/neuroimaging perspective. Neuroscience Journal, 2014, 585237. 10.1155/2014/585237, 26317109 PMC4437268

[bib108] Schwartz, R. G., Scheffler, F. L. V., & Lopez, K. (2013). Speech perception and lexical effects in specific language impairment. Clinical Linguistics & Phonetics, 27(5), 339–354. 10.3109/02699206.2013.763386, 23635335 PMC4058711

[bib109] Scott, T. L., Gallée, J., & Fedorenko, E. (2017). A new fun and robust version of an fMRI localizer for the frontotemporal language system. Cognitive Neuroscience, 8(3), 167–176. 10.1080/17588928.2016.1201466, 27386919

[bib110] Sevitz, J. S., Kiefer, B. R., Huber, J. E., & Troche, M. S. (2021). Obtaining objective clinical measures during telehealth evaluations of dysarthria. American Journal of Speech-Language Pathology, 30(2), 503–516. 10.1044/2020_AJSLP-20-00243, 33689471 PMC8740561

[bib111] Shafer, V. L., Ponton, C., Datta, H., Morr, M. L., & Schwartz, R. G. (2007). Neurophysiological indices of attention to speech in children with specific language impairment. Clinical Neurophysiology, 118(6), 1230–1243. 10.1016/j.clinph.2007.02.023, 17452008 PMC2020430

[bib112] Shain, C., & Fedorenko, E. (2025). A language network in the individualized functional connectomes of over 1,000 human brains doing arbitrary tasks. bioRxiv. 10.1101/2025.03.29.646067, 42595752 PMC13473096

[bib113] Smith, S. M. (2002). Fast robust automated brain extraction. Human Brain Mapping, 17(3), 143–155. 10.1002/hbm.10062, 12391568 PMC6871816

[bib114] Soriano-Mas, C., Pujol, J., Ortiz, H., Deus, J., López-Sala, A., & Sans, A. (2009). Age-related brain structural alterations in children with specific language impairment. Human Brain Mapping, 30(5), 1626–1636. 10.1002/hbm.20620, 18781595 PMC6870989

[bib115] Stoodley, C. J., Valera, E. M., & Schmahmann, J. D. (2012). Functional topography of the cerebellum for motor and cognitive tasks: An fMRI study. NeuroImage, 59(2), 1560–1570. 10.1016/j.neuroimage.2011.08.065, 21907811 PMC3230671

[bib116] Szaflarski, J. P., Gloss, D., Binder, J. R., Gaillard, W. D., Golby, A. J., Holland, S. K., Ojemann, J., Spencer, D. C., Swanson, S. J., French, J. A., & Theodore, W. H. (2017). Practice guideline summary: Use of fMRI in the presurgical evaluation of patients with epilepsy: Report of the Guideline Development, Dissemination, and Implementation Subcommittee of the American Academy of Neurology. Neurology, 88(4), 395–402. 10.1212/WNL.0000000000003532, 28077494 PMC5272968

[bib117] Tallal, P., & Piercy, M. (1974). Developmental aphasia: Rate of auditory processing and selective impairment of consonant perception. Neuropsychologia, 12(1), 83–93. 10.1016/0028-3932(74)90030-X, 4821193

[bib118] Thiel, C. M., Zilles, K., & Fink, G. R. (2004). Cerebral correlates of alerting, orienting and reorienting of visuospatial attention: An event-related fMRI study. NeuroImage, 21(1), 318–328. 10.1016/j.neuroimage.2003.08.044, 14741670

[bib119] Tomblin, J. B., Freese, P. R., & Records, N. L. (1992). Diagnosing specific language impairment in adults for the purpose of pedigree analysis. Journal of Speech and Hearing Research, 35(4), 832–843. 10.1044/JSHR.3504.832, 1405540

[bib120] Tomblin, J. B., Records, N. L., Buckwalter, P., Zhang, X., Smith, E., & O’Brien, M. (1997). Prevalence of specific language impairment in kindergarten children. Journal of Speech, Language, and Hearing Research, 40(6), 1245–1260. 10.1044/jslhr.4006.1245, 9430746 PMC5075245

[bib121] Twait, E., Farah, R., & Horowitz-Kraus, T. (2018). Decreased functional connectivity of the salience network during narrative comprehension in children with reading difficulties: An fMRI study. NeuroImage: Clinical, 20, 987–992. 10.1016/j.nicl.2018.10.006, 30316176 PMC6190597

[bib122] Uddin, L. Q., Supekar, K. S., Ryali, S., & Menon, V. (2011). Dynamic reconfiguration of structural and functional connectivity across core neurocognitive brain networks with development. Journal of Neuroscience, 31(50), 18578–18589. 10.1523/JNEUROSCI.4465-11.2011, 22171056 PMC3641286

[bib123] Ullman, M. T., Clark, G. M., Pullman, M. Y., Lovelett, J. T., Pierpont, E. I., Jiang, X., & Turkeltaub, P. E. (2024). The neuroanatomy of developmental language disorder: A systematic review and meta-analysis. Nature Human Behaviour, 8(5), 962–975. 10.1038/s41562-024-01843-6, 38491094

[bib124] Ullman, M. T., Earle, F. S., Walenski, M., & Janacsek, K. (2020). The neurocognition of developmental disorders of language. Annual Review of Psychology, 71, 389–417. 10.1146/annurev-psych-122216-011555, 31337273

[bib125] Ullman, M. T., & Pierpont, E. I. (2005). Specific language impairment is not specific to language: The procedural deficit hypothesis. Cortex, 41(3), 399–433. 10.1016/S0010-9452(08)70276-4, 15871604

[bib126] Verhoeven, J. S., Rommel, N., Prodi, E., Leemans, A., Zink, I., Vandewalle, E., Noens, I., Wagemans, J., Steyaert, J., Boets, B., Van de Winckel, A., De Cock, P., Lagae, L., & Sunaert, S. (2012). Is there a common neuroanatomical substrate of language deficit between autism spectrum disorder and specific language impairment? Cerebral Cortex, 22(10), 2263–2271. 10.1093/cercor/bhr292, 22047968

[bib127] Verly, M., Gerrits, R., Sleurs, C., Lagae, L., Sunaert, S., Zink, I., & Rommel, N. (2019). The mis-wired language network in children with developmental language disorder: Insights from DTI tractography. Brain Imaging and Behavior, 13(4), 973–984. 10.1007/s11682-018-9903-3, 29934818

[bib128] von Koss Torkildsen, J., Dailey, N. S., Aguilar, J. M., Gómez, R., & Plante, E. (2013). Exemplar variability facilitates rapid learning of an otherwise unlearnable grammar by individuals with language-based learning disability. Journal of Speech, Language, and Hearing Research, 56(2), 618–629. 10.1044/1092-4388(2012/11-0125), 22988285 PMC3973537

[bib129] Vydrova, R., Komarek, V., Sanda, J., Sterbova, K., Jahodova, A., Maulisova, A., Zackova, J., Reissigova, J., Krsek, P., & Kyncl, M. (2015). Structural alterations of the language connectome in children with specific language impairment. Brain and Language, 151, 35–41. 10.1016/j.bandl.2015.10.003, 26609941

[bib130] Wechsler, D. (2018). Wechsler Abbreviated Scale of Intelligence–Second Edition [Data set]. APA PsycTests. 10.1037/t15171-000

[bib131] Wiig, E. H., Semel, E., & Secord, W. (2013). Clinical evaluation of language fundamentals (5th ed.). Pearson/PsychCorp.

[bib132] Winstanley, M., Webb, R. T., & Conti-Ramsden, G. (2018). More or less likely to offend? Young adults with a history of identified developmental language disorders. International Journal of Language & Communication Disorders, 53(2), 256–270. 10.1111/1460-6984.12339, 29159847 PMC5888152

[bib133] Woolrich, M. W., Behrens, T. E. J., Beckmann, C. F., Jenkinson, M., & Smith, S. M. (2004). Multilevel linear modelling for FMRI group analysis using Bayesian inference. NeuroImage, 21(4), 1732–1747. 10.1016/j.neuroimage.2003.12.023, 15050594

[bib134] Xu, P., Wang, M., Zhang, T., Zhang, J., Jin, Z., & Li, L. (2024). The role of middle frontal gyrus in working memory retrieval by the effect of target detection tasks: A simultaneous EEG-fMRI study. Brain Structure and Function, 229(9), 2493–2508. 10.1007/s00429-023-02687-y, 37477712

