# Supplementary material for: The Neural Cost of Language Processing: Broader Network Recruitment in Adults With Developmental Language Disorder
Source: Neurobiol Lang (Camb). 2026 Aug 28;7:NOL.a.277. doi: 10.1162/NOL.a.277 (PMC13597105; doi:10.1162/NOL.a.277)
Supplement: Supplementary file 1 [file nol-07-277-s001.docx]

Supplementary Materials

**Table S1.** Regions of significant BOLD activation during the Degraded speech condition. Clusters were identified using cluster-based thresholding (Z > 3.1, cluster significance p < 0.05, corrected). Coordinates (x, y, z) are reported in MNI space, and cluster sizes are given in voxels. The reported p-values (p corr), t-values (peak t), and coordinates reflect the local maximum within each cluster. The brain regions listed within the cluster are based on the probabilistic regions of the Harvard-Oxford atlas. TD = adults with typical language development, DLD = adults with developmental language disorder, Corr = corrected, R = right, L = left.

| **Cluster** | **Voxels** | ***P* (corr)** | **Peak *t*** | | ***x*** | ***y*** | ***z*** | **Brain regions** | |
| --- | --- | --- | --- | --- | --- | --- | --- | --- | --- |
| **TD** |  |  | | | | | | |  |
| 2 | 16,401 | 0.0012 | 9.69 | | 57 | -13 | 2 | R planum temporale, supramarginal gyrus, Heschl’s gyrus, superior temporal gyrus, and middle temporal gyrus, with extension into planum polare, post-central gyrus, and central opercular cortex | |
| 1 | 15,539 | 0.0012 | | 10.7 | -62 | -12 | 5 | L planum temporale, supramarginal gyrus, Heschl’s gyrus, superior temporal gyrus, and middle temporal gyrus, with extension into planum polare, post-central gyrus, and central opercular cortex | |
| **DLD** |  |  | |  |  |  |  |  | |
| 2 | 5,617 | 0.0032 | | 7.32 | -62 | -12 | 5 | L planum temporale, Heschl’s gyrus, superior temporal gyrus, and middle temporal gyrus, with extension into planum polare, post-central gyrus, and central opercular cortex | |
| 1 | 5,341 | 0.0034 | | 7.64 | 55 | -15 | 3 | R planum temporale, Heschl’s gyrus, superior temporal gyrus, and middle temporal gyrus, with extension into planum polare, post-central gyrus, and central opercular cortex | |
| **TD > DLD** |  |  | |  |  |  |  |  | |
| 1 | 2,722 | 0.036 | | 4.79 | -65 | -17 | 6 | L planum temporale and supramarginal gyrus, with extension into the superior temporal gyrus, post-central gyrus, central opercular cortex, and parietal operculum | |

**Table S2.** Group differences in BOLD activation during the Intact speech condition. Clusters were identified using cluster-based thresholding (Z > 3.1, cluster significance p < 0.05, corrected). Coordinates (x, y, z) are reported in MNI space, and cluster sizes are given in voxels. The reported p-values (p corr), t-values (peak t), and coordinates reflect the local maximum within each cluster. The brain regions listed within the cluster are based on the probabilistic regions of the Harvard-Oxford atlas. TD = adults with typical language development, DLD = adults with developmental language disorder, Corr = corrected, R = right, L = left.

| **Cluster** | **Voxels** | ***P* (corr)** | | **Peak *t*** | ***x*** | ***y*** | ***z*** | **Brain regions** | | | | |
| --- | --- | --- | --- | --- | --- | --- | --- | --- | --- | --- | --- | --- |
| **TD** |  |  |  | | | | | |  |  |  |  |
| 2 | 26,823 | 0.001 | | 10.4 | -62 | -11 | 4 | L planum temporale with extension into the superior and middle temporal gyri, temporal pole, planum polare, Heschl’s gyrus, supramarginal gyrus, angular gyrus, central opercular cortex, parietal operculum cortex, frontal orbital cortex and inferior frontal gyrus (pars triangularis portion) | | | | |
| 1 | 18,924 | 0.0014 | | 9.69 | 41 | 22 | -34 | R planum temporale with extension into the superior and middle temporal gyri, temporal pole, planum polare, Heschl’s gyrus, supramarginal gyrus, central opercular cortex, and precentral gyrus | | | | |
| **DLD** |  |  | |  |  |  |  |  | | | | |
| 2 | 9,668 | 0.0008 | | 8.03 | -61 | -12 | 3 | L planum temporale with extension into the superior temporal gyri, temporal pole, and Heschl’s gyrus | | | | |
| 1 | 7,200 | 0.0014 | | 8.29 | 59 | 8 | -16 | R planum temporale with extension into the superior and middle temporal gyri, temporal pole, and Heschl’s gyrus | | | | |
| **TD > DLD** |  |  | |  |  |  |  |  | | | | |
| 1 | 3,406 | 0.0232 | | 5.07 | -56 | -51 | 17 | L angular gyrus with extension into the superior temporal gyrus, planum temporale, and supramarginal gyrus | | | | |
| **DLD > TD** |  |  | |  |  |  |  |  | | | | |
| 1 | 3,541 | 0.021 | | 4.87 | 47 | 38 | 26 | R frontal pole with extension into the middle frontal gyrus | | | | |

**Table S3.** Significant clusters for the Intact > Degraded contrast in the adults with typical language development (TD) and developmental language disorder (DLD). Clusters were thresholded using cluster-based correction (Z > 3.1, cluster significance p < 0.05, corrected). Coordinates (x, y, z) are reported in MNI space, and cluster sizes are given in voxels. The reported p-values (p corr), t-values (peak t), and coordinates reflect the local maximum within each cluster. The brain regions listed within the cluster are based on the probabilistic regions of the Harvard-Oxford atlas. TD = adults with typical language development, DLD = adults with developmental language disorder, Corr = corrected, R = right, L = left.

| **Cluster** | **Voxels** | ***P* (corr)** | | **Peak *t*** | ***x*** | ***y*** | | ***z*** | **Brain regions** | | | | |
| --- | --- | --- | --- | --- | --- | --- | --- | --- | --- | --- | --- | --- | --- |
| **TD** |  |  |  | | | | | |  |  |  |  |  |
| 3 | 29,842 | 0.001 | | 8.15 | -60 | -5 | | -7 | L superior temporal gyrus, extending into middle temporal gyrus, superior temporal sulcus, planum temporale, temporal pole, inferior temporal gyrus, supramarginal gyrus, and angular gyrus | | | |  |
| 2 | 14,188 | 0.0026 | | 6.59 | 44 | 24 | | -28 | R temporal pole extending into the superior temporal gyrus, middle temporal gyrus, Heschl’s gyrus, and planum polare | | | |  |
| 1 | 6,025 | 0.0136 | | 5.89 | 14 | -76 | | -44 | R cerebellum including crus II with extension into crus I, VI, and VIIb | | | |  |
| **DLD** |  |  | |  |  |  | |  |  | | | |  |
| 3 | 7,308 | 0.004 | | 6.63 | -57 | -10 | | 0 | L superior and middle temporal gyri, including portions of the superior temporal sulcus, planum temporale and planum polare | | | |  |
| 2 | 7,237 | 0.004 | | 6.30 | 45 | 26 | | -27 | R temporal pole with extension into the superior and middle temporal gyri | | | |  |
| 1 | 2055 | 0.0346 | | 5.51 | 25 | -15 | | 42 | R pre-motor and medial frontal cortex | | | |  |
| **DLD > TD** |  |  |  | | | | | |  |  |  | |  |
| 7 | 14,937 | 0.0022 | | 5.35 | 5 | 27 | | 27 | Bilateral anterior cingulate gyrus with extension to paracingulate gyrus | | | |  |
| 6 | 14,173 | 0.0024 | | 5.87 | 34 | 61 | | 3 | R frontal pole with extension into frontal orbital cortex, middle frontal gyrus, insular cortex, caudate nucleus, and putamen | | | |  |
| 5 | 6,304 | 0.0092 | | 5.28 | -39 | 13 | -9 | | L insular cortex, with extension into frontal pole, frontal orbital cortex, insular cortex, caudate nucleus, nucleus accumbens, and putamen | | | |  |
| 4 | 4,679 | 0.0158 | | 5.43 | 2 | -21 | 25 | | Bilateral posterior cingulate gyrus | | | |  |
| 3 | 4,536 | 0.0166 | | 5.45 | -21 | 35 | 33 | | L superior frontal gyrus with extension into frontal and middle frontal gyrus | | | |  |
| 2 | 2,996 | 0.0292 | | 4.92 | 16 | -61 | 37 | | R precuneus cortex | | | |  |
| 1 | 2,490 | 0.0412 | | 5.35 | -14 | -70 | 30 | | L precuneus cortex | | | | |

**Table S4.** Significant clusters reflecting positive correlations between activation for the Intact > Degraded condition and scores on the TILLS Sentence/Discourse composite. Scores were modeled as a continuous variable across all participants. Clusters were thresholded using cluster-based correction (Z > 3.1, cluster significance p < 0.05, corrected). Coordinates (x, y, z) are reported in MNI space, and cluster sizes are given in voxels. The reported p-values (p corr), t-values (peak t), and coordinates reflect the local maximum within each cluster. The brain regions listed within the cluster are based on the probabilistic regions of the Harvard-Oxford atlas. TD = adults with typical language development, DLD = adults with developmental language disorder, Corr = corrected, R = right, L = left.

| **Cluster** | **Voxels** | ***P* (corr)** | **Peak *t*** | ***x*** | ***y*** | ***z*** | **Brain regions** | |
| --- | --- | --- | --- | --- | --- | --- | --- | --- |
| **TD & DLD** | | |  |  |  |  |  |  |
| 3 | 29,332 | 0.0008 | 7.91 | -60 | -5 | -7 | L superior temporal gyrus, extending into middle temporal gyrus, superior temporal sulcus, planum temporale, temporal pole, inferior temporal gyrus, supramarginal gyrus, and angular gyrus |  |
| 2 | 15,509 | 0.0026 | 7.54 | 44 | 24 | -28 | R superior temporal pole extending into the superior and middle temporal gyri, Heschl’s gyrus, and planum temporale |  |
| 1 | 5,426 | 0.019 | 5.70 | 12 | -83 | -47 | R cerebellum including crus II, crus I VI, and VIIb |  |
| **DLD Only** | |  |  |  |  |  |  |  |
| 1 | 22,653 | 0.0206 | 7.79 | -56 | -8 | -8 | L superior temporal gyrus extending into the middle temporal gyrus, temporal pole, planum temporale, Heschl’s gyrus, planum polare, inferior frontal gyrus, frontal operculum, thalamus, hippocampus and putamen |  |

Figure S1. Individual TILLS composite standard score profiles for each adult with DLD across the five composite domains: Sound & Word, Sentence & Discourse, Oral, Written, and ID Core. Each panel represents a single participant, with data points color-coded by their quartile ranking within the DLD group for each composite. The gray shaded region represents the mean ± 1 SD of the typically developing (TD) group, and the dashed line indicates the TD mean.

**Figure S2.** Raw score distributions for TD (green) and DLD (purple) groups across all 17 TILLS subtests. Note that each subtest has a different raw score scale reflecting differences in item number and scoring across subtests.
